# Supplementary material for: What arguments and from whom are most influential in shaping public health policy: thematic content analysis of responses to a public consultation on the regulation of television food advertising to children in the UK
Source: BMJ Open. 2019 Sep 23;9(8):e028221. doi: 10.1136/bmjopen-2018-028221 (PMC6830459; doi:10.1136/bmjopen-2018-028221)
Supplement: Supplementary data [file bmjopen-2018-028221supp001.pdf]

Question 1	Do you agree that the regulatory objectives set out in paragraph 5.2 above are appropriate?
Question 2	Do you consider that it is desirable to distinguish between foods that are high in fat, salt or sugar and those that are healthier in order to achieve the regulatory objectives, or could an undifferentiated approach provide a reasonable alternative?
Question 3	If so, do you consider the FSA's nutrient profiling scheme to be a practical and reasonable basis for doing so? If not, what alternative would you propose? (Note: The nutrient profiling scheme was developed by the FSA and handed to Ofcom following extensive consultation (see FSA web site). This being the case, and given the scheme itself and the science upon which it is based fall outside Ofcom's area of responsibility and expertise, it is not appropriate in this consultation to seek responses on those matters)
Question 4	Do you agree that voluntary self-regulation would not be likely to meet Ofcom's regulatory objectives or the public policy objectives?
Question 5	Do you agree that the exclusion of all HFSS advertising before 9.00pm would be disproportionate?
Question 6	Do you agree that all food and drink advertising and sponsorship should be excluded from programmes aimed at pre-school children?
Question 7	Do you agree that revised content standards should apply to the advertising or sponsorship of all food and drink advertisements?
Question 8	Do you consider that the proposed age bands used in those rules aimed at preventing targeting of specific groups of children are appropriate?
Question 9	Do you consider the proposed content standards including their proposed wording to be appropriate, and if not, what changes would you propose, and why?
Question 10	Do you consider a transitional period would be appropriate for children's channels in the context of the scheduling restrictions, and if so, what measure of the 'amount' of advertising should be used?
Question 11	Do you consider there is a case for exempting low child audience satellite and cable channels from the provisions of Package 3?
Question 12	Do you agree that there should not be a phase-in period for children's channels under Package 3?
Question 13	Which of the three policy packages would you prefer to be incorporated into the advertising code and for what reasons?
Question 14	Alternatively, do you consider that a combination of different elements of the three packages would be suitable? If so, which elements would you favour within an alternative package? (You should note that the analysis in the Impact Assessment has focused on estimating the costs of restricting scheduling, volume, and content separately and would therefore allow consideration of other combinations of the same elements).
Question 15	Where you favour either Package 1 or 2, do you agree that it would be appropriate to allow children's channels a transitional period to phase in restrictions on HFSS / food advertising, on the lines proposed?
Question 16	Do you consider that the packages should include restrictions on brand advertising and sponsorship? If so, what criteria would be most appropriate to define a relevant brand? If not, do you see any issue with the prospect of food manufacturers substituting brand advertising and sponsorship for product promotion?
Question 17	Ofcom invites comments on the implementation approach set out in paragraph 5.45 and 5.46.
