# Supplementary material for: What arguments and from whom are most influential in shaping public health policy: thematic content analysis of responses to a public consultation on the regulation of television food advertising to children in the UK
Source: BMJ Open. 2019 Sep 23;9(8):e028221. doi: 10.1136/bmjopen-2018-028221 (PMC6830459; doi:10.1136/bmjopen-2018-028221)
Supplement: Supplementary data [file bmjopen-2018-028221supp002.pdf]

Advertising stakeholders	Broadcast stakeholders	Lawyers	Politicians	Retailers
Institute of Practitioners in Advertising	Producer's Alliance for Cinema and Television 1	Baker and McKenzie LLP	Mary Creagh MP	Sainsbury
Advertising Association	Five		Welsh Assembly	The Co-operative
Incorporated Society of British Advertisers 1	Channel 4		All Party Parlimentary group on Heart Disease	British Retail Consortium 1
Mediavest Manchester	Flextech television		David Amess MP	British Retail Consortium 2
Zenith Optimedia	ITV			
Mindshare	GMTV			
Incorporated Society of British Advertisers 2	Jetix, Nickelodeon and Turner			
	Producer's Alliance for Cinema and Television 2			
	Broadcast Advertising Clearance Centre			
	British Academy of Film and Television Arts			
	Broadcast Committee of Advertising Practice			
