# Supplementary material for: What arguments and from whom are most influential in shaping public health policy: thematic content analysis of responses to a public consultation on the regulation of television food advertising to children in the UK
Source: BMJ Open. 2019 Sep 23;9(8):e028221. doi: 10.1136/bmjopen-2018-028221 (PMC6830459; doi:10.1136/bmjopen-2018-028221)
Supplement: Supplementary data [file bmjopen-2018-028221supp003.pdf]

Food companies	Food industry representative groups	Civil society groups	Public health stakeholders
Kellogg 1	Food Advertising Unit 1	Kids Inc	National Heart Forum 1
Kraft 1	Food and Drink Federation 2	Consumer Council of Northern Ireland	Northern Ireland Chest Heart and Stroke 2
Pepsico	Snack Nut and Crisp Manufacturers Association 1	Children's Food Bill Coalition	UK Public Health Association
GlaxoSmithKline 1	Food Advertising Unit 2	National Consumer Council 1	Royal Society of Health
United Biscuits	Food and Drink Federation 1	Which 1	Consensus Action on Salt and Health
Nestle	Dairy Council	Officer of the Children's Commissioner	Nutrition Society
Cadbury	Snack Nut and Crisp Manufacturers Association 2	School Food Trust 1	Diabetes UK
Ferrero 1	Provision Trade Federation	Trading Standards Institute	Association for the Study of Obesity
Masterfoods 2	British Cheese Board	Which 2	Heart of Mersey 2
Coca-cola 1	Biscuit Cake Chocolate Confectionary Association 1	Welsh Consumer Council	National Oral Health Promotion Group
McDonalds 2	Biscuit Cake Chocolate Confectionary Association 2	Food Aware	Scientific Advisory Committee on Nutrition
Vimto	Dairy UK	Safefood Ireland	British Psychological Society
Wrigley		The Caroline Walker Trust	British Dietetic Association
Wiltshire farm foods		Advisory Committee for England	National Heart Forum 2
Unilever		Voice of the Listener and Viewer 2	British Heart Foundation
GlaxoSmithKline 2		Advertising Advisory Committess	British Medical Association 1
Coca-cola 2		British Nutrition Foundation	Cheshire and Merseyside Public Health Network
Masterfoods 2		Food Ethics Council	Health Protection Agency Northern Ireland
Kraft 2		Voice of the Listener and the Viewer 1	Irish Heart Foundation 1
McDonalds 1		National Consumer Council 2	National Heart Alliance Ireland 1
RHM Group		National Family and Parenting Institute	National Heart Alliance Ireland 2
Kellogg 2		National Union of Teaachers	International Association for the Study of Obesity 1
Ferrero 2		The Nutrition Society	British Medical Association 2
		Children's Food Campaign	Heart of Mersey 1
		Consumer Council	Northern Ireland Chest Heart and Stroke 1
		Barnardos	Irish Heart Foundation 2
		National Children's Bureau	NHS Borders
		Public Voice	Medical Research Council 1
		School Food Trust	British Heart Foundation 2
		Scotland's Commissioner for Young People	Cancer Research UK
		Food Standards Agency	Northern Ireland Chest Heart and Stroke 3
		National Youth Agency	International Association for the Study of Obesity 2
		Advisory Committee for Northern Ireland	Royal College of Physicians
		Food Commission 1	Weight Concern
		Women's Institute 1	British Dental Association
		The Food Commission	Medical Research Council 2
		The Obesity Awareness and Solutions Trust	Joint statement by the British Heart Foundation, Cancer Research UK and Diabetes UK
		National Federation of Women's Institutes 1	Royal College of Nursing
		National Federation of Women's Institutes 2	
