# Supplementary material for: What arguments and from whom are most influential in shaping public health policy: thematic content analysis of responses to a public consultation on the regulation of television food advertising to children in the UK
Source: BMJ Open. 2019 Sep 23;9(8):e028221. doi: 10.1136/bmjopen-2018-028221 (PMC6830459; doi:10.1136/bmjopen-2018-028221)
Supplement: Author's manuscript [file bmjopen-2018-028221.draft_revisions.pdf]

BMJ Open is committed to open peer review. As part of this commitment we make the peer review history of every article we publish publicly available.

When an article is published we post the peer reviewers' comments and the authors' responses online. We also post the versions of the paper that were used during peer review. These are the versions that the peer review comments apply to.

The versions of the paper that follow are the versions that were submitted during the peer review process. They are not the versions of record or the final published versions. They should not be cited or distributed as the published version of this manuscript.

BMJ Open is an open access journal and the full, final, typeset and author-corrected version of record of the manuscript is available on our site with no access controls, subscription charges or pay-per-view fees (http://bmjopen.bmj.com).

If you have any questions on BMJ Open's open peer review process please email info.bmjopen@bmj.com

BMJ Open

How do different stakeholder groups influence public health policy? Thematic content analysis of responses to a public consultation on the regulation of television food advertising to children in the UK

Journal:	*BMJ Open*
Manuscript ID	bmjopen-2018-028221
Article Type:	Research
Date Submitted by the Author:	27-Nov-2018

Complete List of Authors:	Razavi, Ahmed; Singleton Hospital,<br>Adams, J; University of Cambridge, Centre for Diet & Activity Research<br>White, Martin; Newcastle University, Institute of Health and Society

Keywords:	PUBLIC HEALTH, QUALITATIVE RESEARCH, Health policy < HEALTH SERVICES ADMINISTRATION & MANAGEMENT

SCHOLARONE™
Manuscripts

TITLE PAGE

Article Title: How do different stakeholder groups influence public health policy? Thematic content analysis of responses to a public consultation on the regulation of television food advertising to children in the UK

Authors:

Ahmed Razavi[1] (Academic Clinical Fellow) saar2@mrc-epid.cam.ac.uk, Jean Adams[1] (Senior University Lecturer) jma79@medschl.cam.ac.uk, Martin White (Director of Research[1] and Visiting Professor[2]) martin.white@mrc-epid.cam.ac.uk

1. Centre for Diet & Activity Research, MRC Epidemiology Unit, University of Cambridge, School of Clinical Medicine, Cambridge, CB2 0QQ.
2. Institute of Health & Society, University of Newcastle upon Tyne, Newcastle NE2 4AX.

Corresponding author: Ahmed Razavi – ahmed.razavi@nhs.net

Key words: public health; health policy; diet; qualitative research; influence

4570 words

Competing interests - None

Funding - This work was undertaken by the Centre for Diet and Activity Research (CEDAR), a UKCRC Public Health Research Centre of Excellence. Funding from Cancer Research UK, the British Heart Foundation, the Economic and Social Research Council, the Medical Research Council, the National Institute for Health Research, and the Wellcome Trust, under the auspices of the UK Clinical Research Collaboration, is gratefully acknowledged. The funders had no role in the study design, data collection, analysis, interpretation, or writing, nor in the decision to submit the article for publication.

For peer review only - http://bmjopen.bmj.com/site/about/guidelines.xhtml

Abstract

Objectives: We explore one aspect of the decision-making process - public consultation on policy proposals by a national regulatory body - aiming to understand how public health policy development is influenced by different stakeholders.

Design: We use thematic content analysis to explore responses to a national consultation on the regulation of television advertising of foods high in fat, salt and sugar aimed at children.

Setting: United Kingdom.

Results: 139 responses from key stakeholder groups were analysed to determine how they influenced the regulator's initial proposals for advertising restrictions. The regulator's priorities were questioned throughout the consultation process by public health stakeholders. The eventual restrictions implemented were less strict in many ways than those originally proposed. These changes appeared to be influenced most by commercial, rather than public health, stakeholders.

Conclusions: Public health policy-making may prioritise commercial over public health interests. Tactics such as the questioning and reframing of scientific evidence may be used. In this example exploring the development of policy regulating television food advertising to children, commercial considerations appear to have led to a watering down of initial regulatory proposals. This seems likely to have compromised the ultimate public health effectiveness of the regulations eventually implemented.

Article Summary – Strengths and limitations of this study

- We explore one aspect of the policy making process, namely an Ofcom stakeholder consultation over television advertising restrictions on high fat, salt and sugar foods.
- Established qualitative methodology was used to evaluate all stakeholder responses to this consultation allowing us to identify arguments used in making both pro- and anti-restriction arguments.
- Policy-making can be influenced through other non-public means. Therefore, we are unable to comment on how other methods of influencing policy-making may have affected this consultation's outcome.

For peer review only

For peer review only - http://bmjopen.bmj.com/site/about/guidelines.xhtml

Background

The commercialisation of food has led to changes in our dietary habits.[1] This, combined with more sedentary lifestyles has resulted in a large increase in the burden of obesity and non-communicable diseases.[2] Foods high in fat, salt and sugar (HFSS) are a contributing factor to increasing rates of non-communicable disease[3] and have therefore become a target for public health action.[4] The World Health Organization (WHO) has encouraged member states to take action on non-communicable diseases, including through regulation of the advertising of HFSS foods.[5] However, a 2016 study found that no member states had implemented comprehensive legislation or enforced mandatory regulations regarding marketing of unhealthy food and beverages to young people,[6] despite multiple systematic reviews and journal articles demonstrating how food marketing contributes to childhood obesity.[7–9] Industry groups often seek to influence public health policy.[10] For example, in 2003 a WHO recommendation suggested people should reduce their sugar intake. This resulted in the Sugar Association (a sugar industry information group) pressing the US Congress to cut WHO funding.[11]

Influences on public health policy regarding food are not limited to the food industry. Health professionals, charities, politicians and members of the public have all attempted to influence public health policy making through directly lobbying policy makers and running publicity campaigns in order to influence public opinion. Evidence of the impact of these activities is hard to find in peer-reviewed literature.

Systematic reviews [12,13,14] have demonstrated how the alcohol and tobacco industries focus on lobbying efforts and promoting self-regulation as means to minimise the impact of public health policy on commercial activities. A recent South African study exploring how the policy around alcohol marketing was formulated demonstrated the strategic use of evidence and how commercial and financial interests use influence to avoid regulations.[15] These tactics have also been seen in relation to food where, in one case study, government opinion reflected industry rather than public health opinion.[16] However, at present, we have limited insight into how stakeholders other than those representing industry interests attempt to influence public health policy in general or dietary public health policy in particular. Identifying strategies and arguments used by these interested parties in a public setting may

For peer review only

help inform how public health policy is determined and how it might more effectively be developed in the future.

Policy context

In December 2003, the UK Government asked Ofcom (the UK communications industry regulator) to consider proposals for strengthening rules on television advertising of food aimed at children. (Fig 1). Ofcom decided to use the Food Standards Agency's Nutrient Profiling Model to determine which foods were classified as HFSS. Ofcom originally put three proposed 'packages' of regulations to public consultation in March 2006 (Packages 1-3 in Table 1). Following this, Ofcom produced an alternative package of restrictions (Modified Package 1 in Table 1) in November 2006, on which Ofcom again consulted.

Figure 1: A timeline of the Ofcom process on developing new recommendations for limiting television food advertising to children.

Table 1: Packages of regulations proposed by Ofcom in the initial consultation (March 2006)

**Options**	**Detail**
Package 1	• No HFSS* food advertising during programmes specifically made for children<br>• No HFSS food advertising during programmes of particular appeal to children+ aged 4-9 years
Package 2	• No food or drink advertising during programmes made specifically for children or of particular appeal to children aged up to 9 years
Package 3	• Volume of food and drink advertising to be limited at times when children are most likely to be watching
Modified Package 1	• As per package 1 except restrictions on HFSS food advertising to be extended to programmes of particular appeal to children aged 4-15 years

For peer review only - http://bmjopen.bmj.com/site/about/guidelines.xhtml

* 'Interested parties' are stakeholder groups who may have been affected by the proposed changes, including advertising agencies, advocacy groups, broadcasters, charities, healthcare associations, politicians, the food industry and the general public.

Following the second consultation (November 2006), modified package 1 was recommended by Ofcom and was implemented from January 2009. A comparison of the final regulations implemented to the initial packages proposed suggests that the consultations had substantial impacts on policy decisions. The only independent evaluation of the regulations eventually implemented found no change in the proportion of advertisements seen by children that were for HFSS foods from before to after implementation.[17,18] A '9pm watershed' (i.e. no advertising of HFSS foods before 21.00hr) is now the preferred option of many civil society organisations[19] as well as Public Health England[20] and was a manifesto pledge by the Labour party for the 2017 general election.[21] The recently released Childhood Obesity Plan Chapter 2 also proposes a 9pm watershed.[22]

The consultations on the Ofcom regulations on the restriction of television food advertising to children offers an opportunity to analyse responses from a range of stakeholder groups to a consultation on an important policy that aims to promote dietary public health through regulation of the food industry. We aimed to identify which arguments, and from whom, appeared to be most influential in shaping the changes in Ofcom's position from the initial consultation to the final recommendations.

Methods

We followed the Standards for Reporting Qualitative Research[23] in reporting our findings.

Patient and Public Involvement

This study did not involve use of patient identifiable data and only used publicly-available responses from stakeholder groups.

Data Sources

We qualitatively analysed all written responses from stakeholder groups to the 2006-7 Ofcom public consultation on the regulation of television advertising of food and drink to children. Responses were freely available on the Ofcom website[24] and responses to both the first and second consultations were included. Responses from individual members of the

For peer review only

public were not included as they tended to be very brief and non-specific. We therefore focused our analysis on key stakeholder organisations representing key constituencies. Where needed, Optical Character Recognition software was used to transcribe the responses. The consultation questions can be seen in Table A in the Appendix.

Data Analysis

Conventional thematic content analysis[25] was used to analyse the data and the Framework method[26] used to organise and chart data. This method involves creating coding categories directly from the data and organising coding within a flexible matrix, which can then be adjusted as more codes emerge from the text. As existing literature on the topic of stakeholder influence on public health policy is limited, rather than using preconceived categories with which to code the data, a new framework for analysis was developed, based on no *a priori* assumptions. After familiarisation with the data, coding was performed line by line for each of the responses from interested parties in NVivo (software developed by QSR International for qualitative research).

Each response was assigned to a category based on the organisation from which it originated to stratify responses between the various types of interested parties (Table 2). The longest and second longest submissions from each category were then coded to develop the initial framework.

Table 2: The categories into which stakeholder groups were classified. A list of each group classified by category can be found in the Appendix.

**Category**	**Definition**
Advertising stakeholders	Advertising companies and representative bodies
Broadcast stakeholders	Broadcasting companies and representative bodies
Civil society groups	Groups that represent the interests of all or some of the general population. This does not include groups that may have

For peer review only

	affiliations with industry who would be included in one of the 'stakeholders' groups.
Food manufacturers	Companies that produce and sell food to retailers
Food retailers	A company that sells food to the general population
Food industry representative groups	Bodies that represent the interests of groups of food manufacturers and retailers
Politicians	Persons professionally involved in politics
Public health stakeholders	Groups that focus on promoting the health of the population

Following coding of the first two longest responses by in each category by AR, a set of codes to apply to further responses was agreed between all authors. Codes were also grouped into themes at this stage to provide the most meaningful thematic coding of the data. The remaining responses were all coded using this analytical framework by AR with additional codes being created when needed. Once each of the responses was coded, a 10% sample of the data were independently duplicate-coded by one of the other authors (JA or MW) in order to ensure appropriate categorisation of the various codes and code hierarchy, and to improve internal validity. Using a matrix, the data were charted resulting in a summary of the data by category from each transcript. Illustrative quotations were highlighted at this point.

The resulting charted data were then interpreted and analysed to determine recurrent themes or topics. These were explored further using quotations to demonstrate the range of opinions in relation to each theme or topic. The positions taken by the interested parties were then compared to Ofcom's starting position and final statement, to identify which positions from which stakeholders appeared to have held the most influence on Ofcom's final position.

For peer review only

For peer review only - http://bmjopen.bmj.com/site/about/guidelines.xhtml

Ethics

Ethical permission was not sought for this study. The consultation responses used have been made freely available on the Ofcom website with the full knowledge of their authors. We, therefore, treat this as publically available data which does not require ethical permission for analysis. As we did not seek informed consent from the authors of consultation responses, we do not name them here – although names were provided on the Ofcom website. Instead, we have used only the categories described in Table 2 to identify quotations in our results. This also avoided the study from becoming too focused on specific stakeholder groups rather than building a general picture of arguments used by different stakeholder groups.

Results

Of 1136 responses received to both rounds of consultation, 997 were from individual members of the public (and thus excluded from the analysis); 139 were from stakeholder groups and were included in the analysis; 114 were responses to the initial consultation and 25 responses to the second consultation. The vast majority of responses from individuals were one-line statements of support for some form of restrictions without directly addressing specific issues concerning implementation. As such it was determined that there was not sufficient detail to determine arguments used, or positions taken. Therefore, these responses are unlikely to have influenced Ofcom other than to reaffirm that there was public support for some form of restriction.

The stakeholder responses varied in length from a few lines to double-digit numbers of pages. Most took the form of an initial broad statement outlining a policy position with supporting evidence, followed by shorter responses directed at addressing the specific questions in the consultation as outlined by Ofcom (shown in Table A, Appendix).

For peer review only - http://bmjopen.bmj.com/site/about/guidelines.xhtml

The organisations in the stakeholder groups outlined in Table 2 broadly fell into two separate categories. Civil society groups, politicians and public health stakeholders were encouraging of restrictions in order to reduce the exposure of children to advertising of HFSS foods. Advertising stakeholders, broadcast stakeholders, food manufacturers, food retailers and food industry stakeholders argued that restrictions would minimally impact childhood obesity whilst having a substantial impact on businesses. Though there were subtleties within each group with regards to what level of restrictions would be ideal, there were not sufficient differences in order to further analyse the differences in responses of the various stakeholder groups beyond these two broad categories.

The key changes from the initial Ofcom position to the final recommendations are summarised in Table 3. Arguments relating to each of the principles below, as outlined in the recommendations, were captured from the framework and are described in detail.

Table 3: The changes in Ofcom's position during the course of the consultation

**Initial options presented by Ofcom**	**Consultation responses and Ofcom's reaction**	**Ofcom's final position**	**Reference in consultation**
Ofcom's packages 1-3 varied on 3 key principles:			
1. Restrictions on advertising of all foods versus just HFSS foods	Following the first consultation it was clear that the majority of responses preferred restricting advertising of only HFSS foods.	The eventual package of restrictions enacted was specific to HFSS foods.	Ofcom Executive Summary 1.12
2. Total ban on food advertising versus volume-based restrictions	Almost all stakeholders did not consider volume based restrictions as being effective at reducing exposure to advertising and this option was dismissed following the first consultation.	There was a total ban enacted on HFSS food advertising in programming 'of particular interest to' children.	Ofcom Executive Summary 1.12

For peer review only

3. Restrictions only on children's channels versus all programmes 'of particular interest' to children, irrespective of channel	Some responses highlighted that children may watch adult TV and a ban on all less healthy food advertising before a 9pm watershed may be more effective than focusing specifically on children's programming. Other responses worried that this would disproportionately impact advertising revenues.	Ofcom rejected the idea of a pre-9pm ban due to concerns about the effect it would have on broadcasters, programming and advertising revenues.	Ofcom Executive Summary 1.12
Further changes that were made:			
Restrictions should apply to children aged 4-9 years	Many responses pointed out that children are legally defined as under 16 years.	The restrictions applied to children aged 4-15 years.	Ofcom Final Statement 4.9
All restrictions should start in April 2007	Children's channels argued that they should be allowed a transitional period as they would be affected financially.	Children's channels were allowed a phased implementation of restrictions, with final implementation by January 2009.	Ofcom Final Statement 5.3/5.4

To which foods should restrictions apply?

There was non-partisan agreement that having a blanket ban on all television food advertising was counter-productive and had the possibility of inadvertently reducing exposure of children to advertisements for healthier products.

For peer review only

Quotes: Should restrictions apply to all foods?

"We do not support any options which would restrict advertising of all foods, including foods such as fruit and vegetables, milk and dairy products. These foods can play an important part in children consuming a balanced diet, and we consider that advertising can play a useful role in educating both parents and children in the ways to achieve this." (Food industry stakeholder)

"[Public health stakeholder] believes that it is desirable to distinguish between healthy and unhealthy foods. We do not believe it would be useful to restrict the advertising of all foods because this would mean manufacturers and retailers would be unable to promote healthy foods, such as fresh fruit and vegetables." (Public health stakeholder)

As the underlying aim of the restrictions was to protect health, preventing the advertising of healthy products would be counter-productive. Stakeholder groups agreed that banning advertisements of all foods would be deleterious to efforts to promote healthy eating and promoting a balanced diet.

Total ban or volume based ban?

The idea of a broad volume based restriction rather than a total ban targeting children's programming was proposed in Package 3 and was nearly universally disliked. Broadcasters, advertisers and food industry stakeholders argued that a volume-based restriction would have a very large effect on commercial revenues, whereas public health stakeholders and civil society groups cited how little a volume-based restriction would actually reduce the exposure of children to HFSS food advertising.

Quotes: Would a volume-based restriction be effective?

"The least acceptable option would be Package 3, which would have a devastating effect on our overall revenues - several times greater than Ofcom has estimated – while delivering a

For peer review only - http://bmjopen.bmj.com/site/about/guidelines.xhtml

smaller reduction in the number of times children see food and drink adverts." (*Broadcast* stakeholder)

"Package 3 not only restricts the option to promote healthy foods to children, but also fails to restrict HFSS adverts during periods of viewing when many children are still watching i.e. up to 9pm." (Public health stakeholder)

Many responses argued that Package 3 would result in very little change in exposure of children to television advertising of HFSS foods but would substantially impact broadcasters and advertisers financially. Arguments concerning commercial impacts were used throughout the responses of industry groups, with emphasis on the fact that as a broadcast regulator, Ofcom has a duty to minimise impact on revenues for broadcasters.

Restrictions on children's programming or a pre-9pm watershed ban?

Although not included in any of Ofcom's proposals, civil society groups and public health stakeholders called for restricting all HFSS food advertising before a 9pm 'watershed' (Box 3). Advertisers, broadcasters and the food industry claimed such restrictions would impinge upon adult viewing. All three groups highlighted the trade-off between protecting children and the loss of advertising exposure to adults. Advertisers, broadcasters and food industry groups cited the negative commercial impacts of a pre-9pm watershed ban as outweighing any 'marginal' public health benefits; whereas civil society groups and public health groups saw the public health benefit of a pre-9pm watershed ban as outweighing commercial impacts.

Quotes: Arguments pertaining to the pre-9pm watershed ban on HFSS food advertising

"[Food industry stakeholder organisation] welcomes Ofcom's rejection of the pre-9pm watershed, as this would have been tantamount to a complete ban on the advertising of food and soft drink products on television, and would have impacted on adult airtime." (Food industry stakeholder)

For peer review only

For peer review only - http://bmjopen.bmj.com/site/about/guidelines.xhtml

"We believe that the most suitable option is the pre 9pm ban of HFSS advertising, for the following reasons:

• achieves one of the key regulatory objectives, that of significantly reducing the impact of HFSS advertising on younger children

• removes 82% of the recorded HFSS advertising impacts on all children (aged 4-15)

• contributes substantially to enhancing protection for older children by reducing their exposure to HFSS advertising

•offers the greatest social and health benefits of all options – in the ranges of £50 million - £200 million per year or £250million - £990 million per year (depending on the value of life measure)". (Civil society group)

"The avoidance of intrusive regulation of advertising during adult airtime is only justifiable once full account has been taken to address the over-riding priority to protect children's health. At times when adults and children are watching, the need to protect children must take priority." (Public health stakeholder)

Ofcom rejected banning HFSS food advertising before a 9pm watershed due to the effect this was expected to have on adult viewing times and commercial revenues, suggesting that industry arguments were more persuasive on this topic. Industry groups successfully argued that adult viewing should be unaffected despite the possibility that both children and adults may be watching television together. The need to protect the right of adults to see whatever they wish was a common argument against restricting advertising on television channels that were not explicitly targeted at children. The individual freedom of an adult therefore appeared to be given precedence over exposing children to HFSS food advertising.

Ofcom's research showed that 48% of parents supported restricting HFSS food advertising before 9pm, which was often cited by industry responses as evidence of a lack of public support. Some responses highlighted the fact that the complete figures were 48% in support of a pre-9pm watershed ban, 24% against the ban, with the remainder undecided. An apparently valid complaint made by public health groups regarding this issue was that

For peer review only

For peer review only - http://bmjopen.bmj.com/site/about/guidelines.xhtml

Ofcom did not ever consult on a pre-9pm watershed ban despite its own research showing this would reduce the exposure of children to HFSS advertising by 82%.

We are also able to see here the use of evidence-based arguments by the civil society group in making their case. Often civil society groups and public health stakeholders would cite evidence to support their argument. Food industry representative groups on the other hand tended to cite a lack of evidence and sought to downplay the existing evidence.

To what ages of children should the restrictions apply?

Ofcom initially planned to restrict advertisements targeted at children aged 4-9 years. Under 4s were thought to have little influence over what foods and drinks were given to them and therefore not considered as part of the restrictions. Throughout the consultation food industry representative groups and food manufacturers argued that restricting advertisements to children aged 4-9 was appropriate, whereas as public health stakeholders argued that this should be expanded to cover children aged 4-15 years (Box 4).

Quotes: Arguments pertaining to the age of children to which restrictions should apply

"It is neither logical nor is there any explanation as to why Ofcom should propose to limit the focus of regulation to children aged under 10. The government asked Ofcom to consider proposals for strengthening its rules on television advertising of food to children. It did not ask Ofcom to limit its focus to any particular age group. Ofcom should logically apply restrictions according to its own definition of children (aged 15 [or under])." (Public health stakeholder)

"Children develop and refine their ability to interpret advertising messages as they get older. Existing studies suggest that by 10 years old (indeed, most studies suggest an even earlier age) they are considered to have sufficient cognitive development to understand the implications of television advertising." (Food manufacturer)

For peer review only

For peer review only - http://bmjopen.bmj.com/site/about/guidelines.xhtml

"We are alarmed by the decision to extend volume and scheduling restrictions of food and drink advertising to children under 16. The intention of Ofcom and the government has always been to protect younger children and industry responded on this basis. Ofcom has previously stated that it wished to find a proportionate solution and we question the evidence base on which this decision was made. A review of Ofcom's own literature would seem to contradict the question put to consultation and support the conclusion that young people are capable of differentiating between programming and advertising." (Food industry representative group)

The logic of defining children as aged 4-9 years was questioned by many stakeholders as, according to Ofcom and in the UK, children are legally defined as those under the age of 16 years. A number of food manufacturers stated that they already did not advertise their products to children under 8-12 years. They argued that during adolescence children become 'media literate' and are able to understand advertising and should therefore not be a target of the restrictions.

Industry arguments appeared to suggest that media 'illiterate' children need protecting from HFSS food advertising whereas public health groups suggested all children needed protecting regardless of how 'media literate' they are. Public health groups argued that adolescents are still susceptible to advertising, have more purchasing power and greater pester power than younger children, and may not appreciate the health implications of a poor diet. Ofcom concluded that expanding restrictions to include children aged 4-15 was appropriate.

When should the restrictions start?

The need for a transitional period was also hotly debated (Box 5). Public health stakeholders and civil society groups suggested that as companies were already aware that restrictions were due to be enforced any transitional period should be minimal. Industry groups argued that a transition period was necessary to allow adjustments to be made.

For peer review only - http://bmjopen.bmj.com/site/about/guidelines.xhtml

Quotes: Arguments pertaining to the need for a transitional period

"We do not believe [a] transitional period is appropriate. The arguments for "phasing in" restrictions appear to be of a commercial nature and not supportive of the policy's public health objectives." (Public health stakeholder)

"We would ask for a transitional period of at least three years. This would allow production companies to adjust, and the growing number of public companies to issue profit warnings where necessary." (Broadcast stakeholder)

Instead of starting restrictions soon after announcement of the final policy statement (February 2007), a phased transition over 1-2 years was implemented (varying for different channel types), suggesting industry arguments held more weight on this point. Despite the stated objective of minimising the exposure of children to HFSS food advertising, it appears that Ofcom was more concerned about the potential commercial impact of advertising restrictions and delayed enforcement of the restrictions as a result.

For peer review only

Discussion

Summary of principal findings

This study presents a unique opportunity for a detailed analysis of responses to a public consultation on a public health policy in the UK. Such data is often not in the public domain and these data therefore offer a rare opportunity for scientific scrutiny. For example, responses to the 2016 consultation on the UK Soft Drinks Industry Levy, have not been released. Our paper highlights how, despite the relative transparency of the 2006-7 consultation, policy appeared to be substantially influenced, most importantly by commercial stakeholders.

As far as we are aware, this is the first analysis to examine how a range of stakeholder groups influenced the development of a public health policy aiming to regulate food industry advertising. Ofcom's decision to implement Modified Package 1 contained concessions to commercial as well as civil society and public health stakeholders. However, ultimately industry arguments appeared to hold more sway, with the main concession to public health groups being expanding restrictions to children aged 4-15. For the most part, Ofcom appeared to make concessions to industry arguments. Ofcom appeared to believe that the commercial impact of the regulation of advertising should carry greatest weight, even when the aim of the regulation was to protect children's health. As such, Ofcom rejected a pre-9pm ban, as proposed by public health and civil society stakeholders, instead approving a two year transition period and emphasising the need for 'proportionate action'. Some public health advocates argued that Ofcom, being a broadcast regulator rather than a public health stakeholder, felt an obligation to protect industry interests. The case for restricting advertising was made in a Department of Health 'white paper' (NHS Strategy documents are known as 'white papers'). However, Ofcom was tasked with how to implement these restrictions. Under the Communications Act 2003, Ofcom retains direct responsibility for advertising scheduling policy. This then begs the question of whether a governmental body with a duty to protect broadcasting interests should be leading on public health legislation.

For peer review only - http://bmjopen.bmj.com/site/about/guidelines.xhtml

This conflict between Ofcom's duties to the public and to broadcasters, may have resulted in eventual restrictions that did not appear to alter the level of exposure of children to HFSS food advertising.[17,18] Ofcom appeared to balance arguments related to commercial and public interests, in terms of jobs and the wider economy, with those relating to public health. Being proportionate in their restrictions was frequently cited by Ofcom in their decision making. Ofcom did not, however, appear to consider the cost to the economy of poor health that could stem from a lack of appropriate restrictions. Ofcom also appeared to give greater priority to allowing advertisers access to adults than to restricting exposure to HFSS food advertising among children, who may be viewing the same programming . Industry representative groups tended to highlight commercial arguments whilst citing evidence that appeared to downplay the role of television advertising in childhood obesity. Self-regulation was also touted as an effective measure to address childhood obesity instead of government-mandated regulations. A recent Canadian study showed how self-regulation had limited impact on how much children are exposed to unhealthy food advertising, concluding mandatory regulations were necessary.[27] Public health groups emphasised that the health of children should outweigh any financial concerns and pointed out that even small changes to advertising at an individual level would affect large numbers of children and so accrue to large population level benefits.

Strengths and Limitations

Using established qualitative methods allowed us to identify key themes in the consultation responses according to stakeholder interests. The creation of a *de novo* framework minimised bias that might have been imposed by using a pre-existing framework. Instead, we allowed categories to emerge from the data. The classification of the responses also enabled us to see what positions were taken by the various stakeholders and which type of responses carried the most influence. Measures were taken to maximise the reliability of our coding, such as duplicate coding a sample of consultation responses. The use of publicly available data was resource efficient. Additionally, the use of all the available data ensured that no perspectives were omitted, adding to internally validity. The omission of responses from individual members of the public was because most public responses lacked detail and were no more than a sentence long. Commercial influences on public health policy are

For peer review only - http://bmjopen.bmj.com/site/about/guidelines.xhtml

unlikely to have changed over the past decade with no changes in lobbying rules or policy making procedures, making it highly likely that our findings from the 2007 consultation are applicable today.

There may be alternative methods by which the public influences policy making, such as by writing to their Member of Parliament. This is a study of only one case of public health policy making and our specific findings may not be generalisable to other aspects of dietary public health policy specifically or public health policy more generally. In this consultation, all members of a stakeholder category were treated as one, though there was some inter-category variation on position. There are also other ways by which interested parties could influence Ofcom, which we were unable to examine in this study. For example, Ofcom gave the option of providing confidential responses which were not available for us to incorporate into our dataset. Other informal lobbying may have occurred. Whether such channels of influence were used or whether similar arguments will have been used privately as were used publicly is unclear.

Relationship to existing knowledge

Some literature exists on the methods by which public health advocates influence policy. In 2006, the New Zealand government held an 'Inquiry into Obesity' in order to determine what could be done to limit increasing obesity rates.[16] Jenkin *et al* found that in three out of four domains examined, the governmental position aligned with that of industry groups, with the exception being nutritional policy in schools. In the other three domains, national obesity strategy, food industry policy, and advertising and marketing policy, the analysis determined that the governmental position allied with industry groups. Much like our study, public health groups were shown to have a limited impact on the eventual policies, with industry arguments proving more influential. An explanation suggested for this was the significance of the food industry to New Zealand's economy, highlighting how considerations outside of public health may importantly shape public health policy. It may be the case that similar factors shaped the eventual restrictions in our case study, despite the appearance of Ofcom wanting to develop 'proportionate restrictions', balancing

For peer review only - http://bmjopen.bmj.com/site/about/guidelines.xhtml

commercial and public health interests. The question of what is proportionate appears to be determined by ideology and how much one feels government's role is to protect health even if it impacts on industry. If this is the case, we must question whether commercial companies can ever be truly motivated to improve health at the possible detriment to their short-term profits. A thematic analysis of alcohol industry documents in Australia[28] concluded that the industry attempted to create an impression of social responsibility whilst promoting interventions that did not affect their profits and campaigning against effective interventions that might affect profits. The *de facto* exemption of commercial stakeholders from bearing the negative external costs of their profitable endeavours (e.g. environmental, social or health impacts) has been widely questioned.[29]

Interpretation and implications of the study

Much of the research undertaken to date on stakeholder influences on public health policy has focused on industry behaviours and practices, whereas in this study we have treated both pro-industry and pro-public health groups equally in our analysis. Our findings suggest that, in the case of the Ofcom consultation on the regulation of TV advertising of foods to children, civil society and public health stakeholders carried less weight than their industry counterparts. Industry groups were apparently successfully able to argue that extensive restrictions would impact upon their commercial revenues, suggesting that their economic arguments importantly influenced the thinking of policy-makers. However, the future (external) costs of treating the potential health implications of HFSS food consumption did not appear to influence policy-making. This may be because any potential cost-savings are long-term and would apply to the health sector, for which Ofcom has no governmental responsibility, whereas the short-term costs would apply to the broadcast sector for which Ofcom is the regulatory body.

Public health advocacy is an activity in which many public health professionals are keen to improve to ensure evidence is translated into policy.[30,31] This study highlights that responding to public health policy consultations alone may not result in policy making favourable to public health and other avenues of influence may also need to be explored.

For peer review only

For peer review only - http://bmjopen.bmj.com/site/about/guidelines.xhtml

Conversely, the change in the definition of children from 4-9 years to 4-15 years demonstrates that there is scope for public health advocates to shape policy should an issue be sufficiently clear and difficult to oppose. A more Machiavellian interpretation would be that to define children as aged 4-9 years at the outset may have been a cynical ploy aimed at ensuring that there was at least some ground to concede to public health stakeholders and distract from the more contentious issues. This is supported by the fact that the definition of children as aged 4-9 years was inherently questionable, given Ofcom's own definition of children as under 16 years, in line with the legal and medical definitions used in the UK. A few companies pointed to their media literacy campaigns as evidence that adolescents can understand advertising as an argument against redefining the scope of these restrictions to children aged 4-15 years. Evidence shows that advertisers simply use different ways to target adolescents,[32] rendering media literacy moot,[33] and suggesting that restrictions are still needed to protect adolescents.

The issue of TV advertising of less healthy foods remains highly politically sensitive and at the top of the public health strategy agenda for obesity.[22] Many UK public health organisations have recently campaigned to ban television advertising of less healthy foods before 9pm (the so-called 9pm watershed).[20,21,34–37] Our analysis of the 2006-7 consultation offers specific insights that could be influential in this ongoing national debate, in the same way as such analyses of historical documents have influenced tobacco control efforts in recent years.[13,38] The Ofcom regulation of television advertising of less healthy foods to children is one of few national public health policies of this sort to have been independently evaluated.[18,39] The independent evaluation found that the introduction of the regulations were not associated with a decrease in children's exposure to less healthy food advertising.[39] Our analysis sheds further light on why and how a regulatory policy that appears to have been ineffective in reducing children's exposure to less healthy food advertising came about. Publishing responses to public consultations in full is a key component of transparent policy making. The UK Treasury's reluctance to make available responses to the Soft Drinks Industry Levy consultation is contrary to this principle.

Further questions and future research

For peer review only - http://bmjopen.bmj.com/site/about/guidelines.xhtml

How policy making is influenced through means other than public consultations should be further studied. Other means of applying political pressure such as political lobbying and having indirect relationships with positions of power are much more opaque and difficult to monitor. Parliamentary records of lobbying activity, copies of internal or leaked documents and registers of MPs interests may all be potential sources of data to explore these issues further. Interviews with former or current employees of policy forming bodies such as Ofcom, as well as other stakeholder groups could also be fruitful. Claims made during this consultation, such as industry claims of needing to issue profit warnings as a consequence of lost revenue from these restrictions, could be analysed. Thematic analysis of further documents such as the responses analysed in this study could provide valuable insight into whether a similar combination of commercial arguments and questioning scientific data is used across different public health policy consultations.

Conclusion

This analysis increases our understanding of how influential some stakeholders are in policy making and provides a framework from which further understanding of the influences on public health policy can be determined. From this case study, we can see that commercial influences on dietary public health policy-making appear to be somewhat greater than the influence of public health stakeholders and may have resulted in compromised legislation. In this case, the potential for commercial impacts of legislation promoting public health appeared to outweigh the anticipated population health benefits in policy decision making.

Authors' contributions - The authors declare that they have no competing interests. Responses were coded by AR with a sub-sample duplicate coded by JA or MW. AR, JA and MW contributed to the manuscript in terms of both writing and editing.

For peer review only

References

1. Fleuret P, Fleuret A. Nutrition, Consumption, and Agricultural Change. *Hum Organ*. 1980;39(3):250-260. doi:10.17730/humo.39.3.53332403k1461480.
2. Jebb SA, Moore MS. Contribution of a sedentary lifestyle and inactivity to the etiology of overweight and obesity: current evidence and research issues. *Med Sci Sports Exerc*. 1999;31(11 Suppl):S534-41. doi:10.1097/00005768-199911001-00008.
3. Global Health Observatory. WHO | Obesity. WHO.
4. WHO global co-ordination mechanism on the prevention and control of noncommunicable diseases (GCM/NCD) POLICY BRIEF: REDUCING THE USE OF SALT IN THE FOOD INDUSTRY TO LOWER SODIUM CONSUMPTION. 2014. http://www.who.int/nmh/ncd-coordination-mechanism/Policybrief34.pdf. Accessed June 28, 2017.
5. WHO | Reducing the impact of marketing of foods and non-alcoholic beverages on children. *WHO*. 2014. http://www.who.int/elena/titles/guidance_summaries/food_marketing_children/en/ . Accessed August 8, 2017.
6. Kraak VI, Vandevijvere S, Sacks G, et al. Policy & practice Progress achieved in restricting the marketing of high-fat, sugary and salty food and beverage products to children. *Bull World Heal Organ*. 2016;94:540-548. doi:10.2471/BLT.15.158667.
7. Raine KD, Lobstein T, Landon J, et al. Restricting marketing to children: Consensus on policy interventions to address obesity. *J Public Health Policy*. 2013;34(2):239-253. doi:10.1057/jphp.2013.9.
8. Harris JL, Pomeranz JL, Lobstein T, Brownell KD. A Crisis in the Marketplace: How Food Marketing Contributes to Childhood Obesity and What Can Be Done. *Annu Rev Public Health*. 2009;30(1):211-225. doi:10.1146/annurev.publhealth.031308.100304.
9. Cairns G, Angus K, Hastings G, Caraher M. Systematic reviews of the evidence on the nature, extent and effects of food marketing to children. A retrospective summary. *Appetite*. 2013;62:209-215. doi:10.1016/j.appet.2012.04.017.
10. WHO | Protecting children from the harmful effects of food and drink marketing.

For peer review only

WHO. 2014. http://www.who.int/features/2014/uk-food-drink-marketing/en/. Accessed June 28, 2017.

11. Sibbald B. Sugar industry sour on WHO report. *CMAJ*. 2003;168(12):1585. http://www.pubmedcentral.nih.gov/articlerender.fcgi?artid=156706&tool=pmcentrez&rendertype=abstract. Accessed December 23, 2015.

12. Savell E, Fooks G, Gilmore AB. How does the alcohol industry attempt to influence marketing regulations? A systematic review. *Addiction*. 2016;111(1):18-32. doi:10.1111/add.13048.

13. Savell E, Gilmore AB, Fooks G, Weishaar H, Gilmore A. How Does the Tobacco Industry Attempt to Influence Marketing Regulations? A Systematic Review. Derrick GE, ed. *PLoS One*. 2014;9(2):e87389. doi:10.1371/journal.pone.0087389.

14. Grüning T, Gilmore AB, McKee M. Tobacco industry influence on science and scientists in Germany. *Am J Public Health*. 2006;96(1):20-32. doi:10.2105/AJPH.2004.061507.

15. Bertscher A, London L, Orgill M. Unpacking policy formulation and industry influence: the case of the draft control of marketing of alcoholic beverages bill in South Africa. *Health Policy Plan*. June 2018. doi:10.1093/heapol/czy049.

16. Jenkin G, Signal L, Thomson G. Nutrition policy in whose interests? A New Zealand case study. *Public Health Nutr*. 2012;15(8):1483-1488. doi:10.1017/S1368980011003028.

17. Adams J, Tyrrell R, Adamson AJ, White M. Effect of restrictions on television food advertising to children on exposure to advertisements for “less healthy” foods: repeat cross-sectional study. *PLoS One*. 2012;7(2):e31578. doi:10.1371/journal.pone.0031578.

18. Adams J, Hennessy-Priest K, Ingimarsdóttir S, Sheeshka J, Ostbye T, White M. Food advertising during children’s television in Canada and the UK. *Arch Dis Child*. 2009;94(9):658-662. doi:10.1136/adc.2008.151019.

19. Children’s Food Campaign. *Through the Looking Glass: A Review of Topsy-Turvy Junk Food Marketing Regulations*.; 2013.

For peer review only

For peer review only - http://bmjopen.bmj.com/site/about/guidelines.xhtml

https://www.sustainweb.org/publications/through_the_looking_glass/. Accessed October 10, 2017.

20. Sugar Reduction The evidence for action. 2015. www.gov.uk/phe. Accessed October 10, 2017.

21. The Labour Party. Labour Manifesto. http://www.labour.org.uk/index.php/manifesto2017. Published 2017. Accessed June 28, 2017.

22. *Childhood Obesity: A Plan for Action Chapter 2 2 DH ID Box Title: Childhood Obesity: A Plan for Action, Chapter 2.*; 2018. www.nationalarchives.gov.uk/doc/open-government-licence/. Accessed November 27, 2018.

23. O'Brien BC, Harris IB, Beckman TJ, Reed DA, Cook DA. Standards for reporting qualitative research: a synthesis of recommendations. *Acad Med*. 2014;89(9):1245-1251. doi:10.1097/ACM.0000000000000388.

24. Ofcom. *Television Advertising of Food and Drink Products to Children: Final Statement*.

25. Hsieh H-F, Shannon SE. Three approaches to qualitative content analysis. *Qual Health Res*. 2005;15(9):1277-1288. doi:10.1177/1049732305276687.

26. Gale NK, Heath G, Cameron E, Rashid S, Redwood S. Using the framework method for the analysis of qualitative data in multi-disciplinary health research. *BMC Med Res Methodol*. 2013;13:117. doi:10.1186/1471-2288-13-117.

27. Potvin Kent M, Wanless A. The influence of the Children's Food and Beverage Advertising Initiative: change in children's exposure to food advertising on television in Canada between 2006-2009. *Int J Obes (Lond)*. 2014;38(4):558-562. doi:10.1038/ijo.2014.4.

28. Miller PG, de Groot F, McKenzie S, Droste N. Vested interests in addiction research and policy. Alcohol industry use of social aspect public relations organizations against preventative health measures. *Addiction*. 2011;106(9):1560-1567. doi:10.1111/j.1360-0443.2011.03499.x.

29. Stuckler D, Nestle M. Big food, food systems, and global health. *PLoS Med*.

For peer review only

For peer review only - http://bmjopen.bmj.com/site/about/guidelines.xhtml

2012;9(6):e1001242. doi:10.1371/journal.pmed.1001242.

30. Dorfman L, Krasnow ID. Public Health and Media Advocacy. *Annu Rev Public Health*. 2014;35(1):293-306. doi:10.1146/annurev-publhealth-032013-182503.

31. Herrick C. The post-2015 landscape: vested interests, corporate social responsibility and public health advocacy. *Sociol Health Illn*. 2016;38(7):1026-1042. doi:10.1111/1467-9566.12424.

32. Livingstone S, Helsper EJ. Does Advertising Literacy Mediate the Effects of Advertising on Children? A Critical Examination of Two Linked Research Literatures in Relation to Obesity and Food Choice. *J Commun*. 2006;56(3):560-584. doi:10.1111/j.1460-2466.2006.00301.x.

33. Montgomery KC, Chester J. Interactive Food and Beverage Marketing: Targeting Adolescents in the Digital Age. *J Adolesc Heal*. 2009;45(3):S18-S29. doi:10.1016/j.jadohealth.2009.04.006.

34. Sustain / Children’s Food Campaign. Junk Food Marketing. https://www.sustainweb.org/childrensfoodcampaign/junk_food_marketing/. Accessed November 1, 2017.

35. Cancer Research UK. Being overweight or obese could cause around 700,000 new UK cancers by 2035 | Cancer Research UK. http://www.cancerresearchuk.org/about-us/cancer-news/press-release/2016-01-07-being-overweight-or-obese-could-cause-around-700000-new-uk-cancers-by-2035. Accessed November 1, 2017.

36. Obesity Health Alliance. Tackling Childhood Obesity - 2017 Election Manifesto. http://obesityhealthalliance.org.uk/policy/. Accessed November 1, 2017.

37. Faculty of Public Health. Start Well, Live Better. http://www.fph.org.uk/start_well%2C_live_better_-_a_manifesto. Accessed November 1, 2017.

38. Fooks GJ, Gilmore AB. Corporate philanthropy, political influence, and health policy. *PLoS One*. 2013;8(11):e80864. doi:10.1371/journal.pone.0080864.

39. Adams J, Tyrrell R, Adamson AJ, White M. Effect of restrictions on television food advertising to children on exposure to advertisements for “less healthy” foods: repeat

For peer review only

cross-sectional study. *PLoS One*. 2012;7(2):e31578. doi:10.1371/journal.pone.0031578.

40. Ofcom. Television Advertising of Food and Drink Products to Children - Ofcom. https://www.ofcom.org.uk/consultations-and-statements/category-2/foodads_new. Accessed August 8, 2017.

For peer review only

For peer review only - http://bmjopen.bmj.com/site/about/guidelines.xhtml

Figure titles and legends

Figure 1: A timeline of the Ofcom process on developing new recommendations for limiting television food advertising to children.

Figure 1 legend:

* 'Interested parties' are stakeholder groups who may have been affected by the proposed changes, including advertising agencies, advocacy groups, broadcasters, charities, healthcare associations, politicians, the food industry and the general public.

Table 1: Packages of regulations proposed by Ofcom in the initial consultation (March 2006)

Table 1 legend:

* HFSS food = High, Fat, Sugar and Salt foods

+ 'of particular appeal to children' = when the proportion of people watching who are children is more than 120% of the proportion of children in the UK population[40]

Table 2: The categories into which stakeholder groups were classified. A list of each group classified by category can be found in the Appendix.

Table 3: The changes in Ofcom's position during the course of the consultation

Appendix

Table A: The questions Ofcom asked as part of the consultation

Table B1: The classification of the responses by organisational category

Table B2: The classification of the responses by organisational category (continued)

For peer review only - http://bmjopen.bmj.com/site/about/guidelines.xhtml

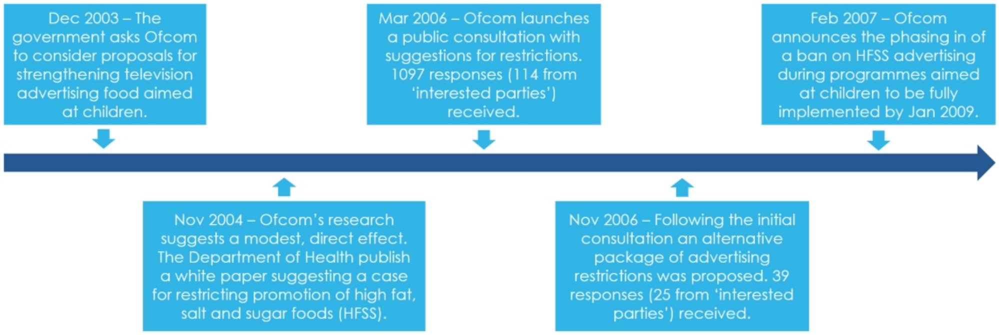

Figure 1: A timeline of the Ofcom process on developing new recommendations for limiting television food advertising to children.

Legend: * 'Interested parties' are stakeholder groups who may have been affected by the proposed changes, including advertising agencies, advocacy groups, broadcasters, charities, healthcare associations, politicians, the food industry and the general public.

Options	Detail
Package 1	• No HFSS* food advertising in programmes specifically made for children<br>• No HFSS food advertising in programmes of particular appeal to children+ aged 4-9 years
Package 2	• No food or drink advertising in programmes made specifically for children or of particular appeal to children aged up to 9 years
Package 3	• Volume of food and drink advertising to be limited at times when children are most likely to be watching
Modified Package 1	• As per package 1 except restrictions on HFSS food advertising to be extended to programmes of particular appeal to children aged 4-15 years

Table 1: Packages of regulations proposed by Ofcom in the initial consultation (March 2006)

Legend: * HFSS food = High, Fat, Sugar and Salt foods
+ 'of particular appeal to children' = when the proportion of people watching who are children is more than 120% of the proportion of children in the UK population[39]

Category	Definition
Advertising interests	Advertising companies and representative bodies
Broadcast interests	Broadcasting companies and representative bodies
Civil society groups	Groups that represent the interests of all or some of the general population
Food manufacturers	Companies that produce and sell food to retailers
Food retailers	A company that sells food to the general population
Food industry interests	Bodies that represent the interests of groups of food manufacturers and retailers
Politicians	Persons professionally involved in politics
Public health interests	Groups that focus on promoting the health of the population

Table 2: The categories into which stakeholder groups were classified. A list of each group classified by category can be found in the Appendix.

Initial options presented by Ofcom	Consultation responses and Ofcom's reaction	Ofcom's final position	Reference in consultation
Ofcom's packages 1-3 varied on 3 key principles:			
1. Restrictions on advertising of all foods versus just HFSS foods	Following the first consultation it was clear that the majority of responses preferred restricting advertising of only HFSS foods.	The eventual package of restrictions enacted was specific to HFSS foods.	Ofcom Executive Summary 1.12
2. Total ban on food advertising versus volume-based restrictions	Almost all stakeholders did not consider volume based restrictions as being effective at reducing exposure to advertising and this option was dismissed following the first consultation.	There was a total ban enacted on HFSS food advertising in programming 'of particular interest to' children.	Ofcom Executive Summary 1.12
3. Restrictions only on children's channels versus all programmes 'of particular interest' to children irrespective of channel	Some responses highlighted that children may watch adult TV and a ban on all less healthy food advertising before the 9pm watershed may be more effective than focusing specifically on children's programming. Other responses worried that this would disproportionately impact advertising revenues.	Ofcom rejected the idea of a pre-9pm ban due to concerns about the effect it would have on broadcasters, programming and advertising revenues.	Ofcom Executive Summary 1.12
Further changes that were made:			
Restrictions should apply to children aged 4-9 years	Many responses pointed out that children are legally defined as under 16 years.	The restrictions applied to children aged 4-15 years.	Ofcom Final Statement 4.9
All restrictions should start in April 2007	Children's channels argued that they should be allowed a transitional period as they would be affected financially.	Children's channels were allowed a phased implementation of restrictions, with final implementation by January 2009.	Ofcom Final Statement 5.3/5.4

Table 3: The changes in Ofcom's position during the course of the consultation

Question 1	Do you agree that the regulatory objectives set out in paragraph 5.2 above are appropriate?
Question 2	Do you consider that it is desirable to distinguish between foods that are high in fat, salt or sugar and those that are healthier in order to achieve the regulatory objectives, or could an undifferentiated approach provide a reasonable alternative?
Question 3	If so, do you consider the FSA's nutrient profiling scheme to be a practical and reasonable basis for doing so? If not, what alternative would you propose? (Note: The nutrient profiling scheme was developed by the FSA and handed to Ofcom following extensive consultation (see FSA web site). This being the case, and given the scheme itself and the science upon which it is based fall outside Ofcom's area of responsibility and expertise, it is not appropriate in this consultation to seek responses on those matters)
Question 4	Do you agree that voluntary self-regulation would not be likely to meet Ofcom's regulatory objectives or the public policy objectives?
Question 5	Do you agree that the exclusion of all HFSS advertising before 9.00pm would be disproportionate?
Question 6	Do you agree that all food and drink advertising and sponsorship should be excluded from programmes aimed at pre-school children?
Question 7	Do you agree that revised content standards should apply to the advertising or sponsorship of all food and drink advertisements?
Question 8	Do you consider that the proposed age bands used in those rules aimed at preventing targeting of specific groups of children are appropriate?
Question 9	Do you consider the proposed content standards including their proposed wording to be appropriate, and if not, what changes would you propose, and why?
Question 10	Do you consider a transitional period would be appropriate for children's channels in the context of the scheduling restrictions, and if so, what measure of the 'amount' of advertising should be used?
Question 11	Do you consider there is a case for exempting low child audience satellite and cable channels from the provisions of Package 3?
Question 12	Do you agree that there should not be a phase-in period for children's channels under Package 3?
Question 13	Which of the three policy packages would you prefer to be incorporated into the advertising code and for what reasons?
Question 14	Alternatively, do you consider that a combination of different elements of the three packages would be suitable? If so, which elements would you favour within an alternative package? (You should note that the analysis in the Impact Assessment has focused on estimating the costs of restricting scheduling, volume, and content separately and would therefore allow consideration of other combinations of the same elements).
Question 15	Where you favour either Package 1 or 2, do you agree that it would be appropriate to allow children's channels a transitional period to phase in restrictions on HFSS / food advertising, on the lines proposed?
Question 16	Do you consider that the packages should include restrictions on brand advertising and sponsorship? If so, what criteria would be most appropriate to define a relevant brand? If not, do you see any issue with the prospect of food manufacturers substituting brand advertising and sponsorship for product promotion?
Question 17	Ofcom invites comments on the implementation approach set out in paragraph 5.45 and 5.46.

Advertising stakeholders	Broadcast stakeholders	Lawyers	Politicians	Retailers
Institute of Practitioners in Advertising	Producer's Alliance for Cinema and Television 1	Baker and McKenzie LLP	Mary Creagh MP	Sainsbury
Advertising Association	Five		Welsh Assembly	The Co-operative
Incorporated Society of British Advertisers 1	Channel 4		All Party Parlimentary group on Heart Disease	British Retail Consortium 1
Mediavest Manchester	Flextech television		David Amess MP	British Retail Consortium 2
Zenith Optimedia	ITV			
Mindshare	GMTV			
Incorporated Society of British Advertisers 2	Jetix, Nickelodeon and Turner			
	Producer's Alliance for Cinema and Television 2			

For peer review only

	Broadcast Advertising Clearance Centre				
	British Academy of Film and Television Arts				
	Broadcast Committee of Advertising Practice				

For peer review only

Food companies	Food industry representative groups	Civil society groups	Public health stakeholders
Kellogg 1	Food Advertising Unit 1	Kids Inc	National Heart Forum 1
Kraft 1	Food and Drink Federation 2	Consumer Council of Northern Ireland	Northern Ireland Chest Heart and Stroke 2
Pepsico	Snack Nut and Crisp Manufacturers Association 1	Children's Food Bill Coalition	UK Public Health Association
GlaxoSmithKline 1	Food Advertising Unit 2	National Consumer Council 1	Royal Society of Health
United Biscuits	Food and Drink Federation 1	Which 1	Consensus Action on Salt and Health
Nestle	Dairy Council	Officer of the Children's Commissioner	Nutrition Society
Cadbury	Snack Nut and Crisp Manufacturers Association 2	School Food Trust 1	Diabetes UK
Ferrero 1	Provision Trade Federation	Trading Standards Institute	Association for the Study of Obesity

For peer review only

Masterfoods 2	British Cheese Board	Which 2	Heart of Mersey 2
Coca-cola 1	Biscuit Cake Chocolate Confectionary Association 1	Welsh Consumer Council	National Oral Health Promotion Group
McDonalds 2	Biscuit Cake Chocolate Confectionary Association 2	Food Aware	Scientific Advisory Committee on Nutrition
Vimto	Dairy UK	Safefood Ireland	British Psychological Society
Wrigley		The Caroline Walker Trust	British Dietetic Association
Wiltshire farm foods		Advisory Committee for England	National Heart Forum 2
Unilever		Voice of the Listener and Viewer 2	British Heart Foundation
GlaxoSmithKline 2		Advertising Advisory Committess	British Medical Association 1
Coca-cola 2		British Nutrition Foundation	Cheshire and Merseyside Public Health Network

For peer review only

Masterfoods 2		Food Ethics Council	Health Protection Agency Northern Ireland
Kraft 2		Voice of the Listener and the Viewer 1	Irish Heart Foundation 1
McDonalds 1		National Consumer Council 2	National Heart Alliance Ireland 1
RHM Group		National Family and Parenting Institute	National Heart Alliance Ireland 2
Kellogg 2		National Union of Teaachers	International Association for the Study of Obesity 1
Ferrero 2		The Nutrition Society	British Medical Association 2
		Children's Food Campaign	Heart of Mersey 1
		Consumer Council	Northern Ireland Chest Heart and Stroke 1
		Barnardos	Irish Heart Foundation 2
		National Children's Bureau	NHS Borders

For peer review only

		Public Voice	Medical Research Council 1
		School Food Trust	British Heart Foundation 2
		Scotland's Commissioner for Young People	Cancer Research UK
		Food Standards Agency	Northern Ireland Chest Heart and Stroke 3
		National Youth Agency	International Association for the Study of Obesity 2
		Advisory Committee for Northern Ireland	Royal College of Physicians
		Food Commission 1	Weight Concern
		Women's Institute 1	British Dental Association
		The Food Commission	Medical Research Council 2

For peer review only

		The Obesity Awareness and Solutions Trust	Joint statement by the British Heart Foundation, Cancer Research UK and Diabetes UK
		National Federation of Women's Institutes 1	Royal College of Nursing
		National Federation of Women's Institutes 2	

For peer review only

Reporting checklist for qualitative study.

Based on the SRQR guidelines.

Instructions to authors

Complete this checklist by entering the page numbers from your manuscript where readers will find each of the items listed below.

Your article may not currently address all the items on the checklist. Please modify your text to include the missing information. If you are certain that an item does not apply, please write "n/a" and provide a short explanation.

Upload your completed checklist as an extra file when you submit to a journal.

In your methods section, say that you used the SRQR reporting guidelines, and cite them as:

O'Brien BC, Harris IB, Beckman TJ, Reed DA, Cook DA. Standards for reporting qualitative research: a synthesis of recommendations. Acad Med. 2014;89(9):1245-1251.

		Reporting Item	Page Number
	#1	Concise description of the nature and topic of the study identifying the study as qualitative or indicating the approach (e.g. ethnography, grounded theory) or data collection methods (e.g. interview, focus group) is recommended	5
	#2	Summary of the key elements of the study using the abstract format of the intended publication; typically	2

For peer review only

		includes background, purpose, methods, results and conclusions	
Problem formulation	#3	Description and signifcance of the problem / phenomenon studied: review of relevant theory and empirical work; problem statement	4
Purpose or research question	#4	Purpose of the study and specific objectives or questions	5
Qualitative approach and research paradigm	#5	Qualitative approach (e.g. ethnography, grounded theory, case study, phenomenolgy, narrative research) and guiding theory if appropriate; identifying the research paradigm (e.g. postpositivist, constructivist / interpretivist) is also recommended; rationale. The rationale should briefly discuss the justification for choosing that theory, approach, method or technique rather than other options available; the assumptions and limitations implicit in those choices and how those choices influence study conclusions and transferability. As appropriate the rationale for several items might be discussed together.	5
Researcher characteristics and reflexivity	#6	Researchers' characteristics that may influence the research, including personal attributes, qualifications / experience, relationship with participants, assumptions and / or presuppositions; potential or actual interaction between researchers' characteristics and the research questions, approach, methods, results and / or transferability	1

For peer review only

Context	#7	Setting / site and salient contextual factors; rationale	4
Sampling strategy	#8	How and why research participants, documents, or events were selected; criteria for deciding when no further sampling was necessary (e.g. sampling saturation); rationale	5
Ethical issues pertaining to human subjects	#9	Documentation of approval by an appropriate ethics review board and participant consent, or explanation for lack thereof; other confidentiality and data security issues	8
Data collection methods	#10	Types of data collected; details of data collection procedures including (as appropriate) start and stop dates of data collection and analysis, iterative process, triangulation of sources / methods, and modification of procedures in response to evolving study findings; rationale	5
Data collection instruments and technologies	#11	Description of instruments (e.g. interview guides, questionnaires) and devices (e.g. audio recorders) used for data collection; if / how the instruments(s) changed over the course of the study	6
Units of study	#12	Number and relevant characteristics of participants, documents, or events included in the study; level of participation (could be reported in results)	8
Data processing	#13	Methods for processing data prior to and during analysis, including transcription, data entry, data management and	6

For peer review only

		security, verification of data integrity, data coding, and anonymisation / deidentification of excerpts	
Data analysis	#14	Process by which inferences, themes, etc. were identified and developed, including the researchers involved in data analysis; usually references a specific paradigm or approach; rationale	6
Techniques to enhance trustworthiness	#15	Techniques to enhance trustworthiness and credibility of data analysis (e.g. member checking, audit trail, triangulation); rationale	7
Syntheses and interpretation	#16	Main findings (e.g. interpretations, inferences, and themes); might include development of a theory or model, or integration with prior research or theory	8
Links to empirical data	#17	Evidence (e.g. quotes, field notes, text excerpts, photographs) to substantiate analytic findings	11
Intergration with prior work, implications, transferability and contribution(s) to the field	#18	Short summary of main findings; explanation of how findings and conclusions connect to, support, elaborate on, or challenge conclusions of earlier scholarship; discussion of scope of application / generalizability; identification of unique contributions(s) to scholarship in a discipline or field	17
Limitations	#19	Trustworthiness and limitations of findings	18
Conflicts of interest	#20	Potential sources of influence of perceived influence on study conduct and conclusions; how these were	1

For peer review only

		managed	
Funding	#21	Sources of funding and other support; role of funders in data collection, interpretation and reporting	1

The SRQR checklist is distributed with permission of Wolters Kluwer © 2014 by the Association of American Medical Colleges. This checklist was completed on 26. November 2018 using http://www.goodreports.org/, a tool made by the EQUATOR Network in collaboration with Penelope.ai

For peer review only

BMJ Open

How do different stakeholder groups influence public health policy? Thematic content analysis of responses to a public consultation on the regulation of television food advertising to children in the UK

Journal:	*BMJ Open*
Manuscript ID	bmjopen-2018-028221.R1
Article Type:	Research
Date Submitted by the Author:	22-Mar-2019

Complete List of Authors:	Razavi, Ahmed; MRC Epidemiology Unit<br>Adams, J; University of Cambridge, Centre for Diet & Activity Research<br>White, Martin; Newcastle University, Institute of Health and Society; University of Cambridge, Centre for Diet & Activity Research

**Primary Subject Heading**:	Public health
Secondary Subject Heading:	Health policy, Public health, Qualitative research
Keywords:	PUBLIC HEALTH, QUALITATIVE RESEARCH, Health policy < HEALTH SERVICES ADMINISTRATION & MANAGEMENT

SCHOLARONE™
Manuscripts

TITLE PAGE

Article Title: How do different stakeholder groups influence public health policy? Thematic content analysis of responses to a public consultation on the regulation of television food advertising to children in the UK

Authors:

Ahmed Razavi[1] (Academic Clinical Fellow) saar2@mrc-epid.cam.ac.uk, Jean Adams[1] (Senior University Lecturer) jma79@medschl.cam.ac.uk, Martin White (Director of Research[1] and Visiting Professor[2]) martin.white@mrc-epid.cam.ac.uk

1. Centre for Diet & Activity Research, MRC Epidemiology Unit, University of Cambridge, School of Clinical Medicine, Cambridge, CB2 0QQ.
2. Institute of Health & Society, University of Newcastle upon Tyne, Newcastle NE2 4AX.

Corresponding author: Ahmed Razavi – ahmed.razavi@nhs.net

Key words: public health; health policy; diet; qualitative research; influence

4570 words

Competing interests - None

Funding - This work was undertaken by the Centre for Diet and Activity Research (CEDAR), a UKCRC Public Health Research Centre of Excellence. Funding from Cancer Research UK, the British Heart Foundation, the Economic and Social Research Council, the Medical Research Council, the National Institute for Health Research, and the Wellcome Trust, under the auspices of the UK Clinical Research Collaboration, is gratefully acknowledged. The funders had no role in the study design, data collection, analysis, interpretation, or writing, nor in the decision to submit the article for publication.

There was no patient or public involvement in the study design or conduct of the study.

Data availability - Data used was freely available from the Ofcom website until a recent update to the website following which data was removed from the website. Ofcom may be able to provide access to the responses on request.

For peer review only - http://bmjopen.bmj.com/site/about/guidelines.xhtml

Abstract

Objectives: We explore one aspect of the decision-making process - public consultation on policy proposals by a national regulatory body - aiming to understand how public health policy development is influenced by different stakeholders.

Design: We use thematic content analysis to explore responses to a national consultation on the regulation of television advertising of foods high in fat, salt and sugar aimed at children.

Setting: United Kingdom.

Results: 139 responses from key stakeholder groups were analysed to determine how they influenced the regulator's initial proposals for advertising restrictions. The regulator's priorities were questioned throughout the consultation process by public health stakeholders. The eventual restrictions implemented were less strict in many ways than those originally proposed. These changes appeared to be influenced most by commercial, rather than public health, stakeholders.

Conclusions: Public health policy-making may prioritise commercial over public health interests. Tactics such as the questioning and reframing of scientific evidence may be used. In this example exploring the development of policy regulating television food advertising to children, commercial considerations appear to have led to a watering down of initial regulatory proposals. This seems likely to have compromised the ultimate public health effectiveness of the regulations eventually implemented.

Article Summary – Strengths and limitations of this study

- Established qualitative methodology (thematic content analysis) was used to evaluate all stakeholder responses.
- A *de novo* analytical framework was created, minimising bias that may have occurred from using a pre-existing framework.
- Stakeholder groups were sorted into eight broad categories allowing us to compare and contrast responses by category.
- Policy-making can be influenced through other non-public means (e.g. direct lobbying), making us unable to comment on how other methods of influencing policy-making may have affected this consultation's outcome.

For peer review only - http://bmjopen.bmj.com/site/about/guidelines.xhtml

- This is one case study of influencing policy and our findings may not be generalisable to other cases.

For peer review only

For peer review only - http://bmjopen.bmj.com/site/about/guidelines.xhtml

Background

Foods high in fat, salt and sugar (HFSS) are a contributing factor to increasing rates of non-communicable disease worldwide[1] and the World Health Organization (WHO) has encouraged member states to take action on non-communicable diseases, including through regulation of the advertising of HFSS foods.[2] However, a 2016 study found that no member states had implemented comprehensive legislation restricting marketing of unhealthy food and beverages to young people,[3] despite multiple systematic reviews demonstrating the importance of food marketing as a driver of childhood obesity.[4–6]

Industry groups often seek to influence public health policy.[7] For example, in 2003 a WHO recommendation suggesting reduction in population sugar intake resulted in the Sugar Association (a sugar industry information group) pressing the US Congress to cut WHO funding.[8] However, influences on dietary public health policy are not limited to the food industry. Health professionals, charities, politicians and members of the public have all attempted to influence policy making. Evidence of the impact of these activities is hard to find in peer-reviewed literature.

Systematic reviews [9,10,11] have demonstrated how the alcohol and tobacco industries focus on lobbying efforts and promote self-regulation as means to minimise the impact of public health policy on commercial activities. These tactics have also been seen in relation to food where, in one case study, government opinion reflected industry rather than public health opinion.[12] However, at present, we have limited insight into how stakeholders other than those representing industry interests attempt to influence public health policy in general or dietary public health policy in particular. Identifying strategies and arguments used by these interested parties in a public setting may help inform how public health policy is determined and how it might more effectively be developed in the future.

Policy context

In December 2003, the UK Government asked Ofcom (the UK communications industry regulator) to consider proposals for strengthening rules on television advertising of food aimed at children (Fig 1). Ofcom decided to use the Food Standards Agency's Nutrient Profiling Model to determine which foods were classified as HFSS. Ofcom originally put

For peer review only - http://bmjopen.bmj.com/site/about/guidelines.xhtml

three proposed ‘packages’ of regulations to public consultation in March 2006 (Packages 1-3 in Table 1). Following this, Ofcom produced an alternative package of restrictions (Modified Package 1 in Table 1) in November 2006, on which Ofcom again consulted.

Figure 1: A timeline of the Ofcom process on developing new recommendations for limiting television food advertising to children.

Table 1: Packages of regulations proposed by Ofcom in the initial consultation (March 2006)

**Options**	**Detail**
Package 1	• No HFSS* food advertising during programmes specifically made for children<br>• No HFSS food advertising during programmes of particular appeal to children+ aged 4-9 years
Package 2	• No food or drink advertising during programmes made specifically for children or of particular appeal to children aged up to 9 years
Package 3	• Volume of food and drink advertising to be limited at times when children are most likely to be watching
Modified Package 1	• As per package 1 except restrictions on HFSS food advertising to be extended to programmes of particular appeal to children aged 4-15 years

* ’Interested parties’ are stakeholder groups who may have been affected by the proposed changes, including advertising agencies, advocacy groups, broadcasters, charities, healthcare associations, politicians, the food industry and the general public.

Following the second consultation (November 2006), modified package 1 was recommended by Ofcom and was implemented from January 2009. A comparison of the final regulations implemented to the initial packages proposed suggests that the consultations had substantial impacts on policy decisions. The only independent evaluation of the regulations eventually implemented found no change in the proportion of advertisements seen by children that were for HFSS foods from before to after

For peer review only

implementation and an increase exposure of HFSS advertising among adults.[13,14] A '9pm watershed' (i.e. no advertising of HFSS foods before 21.00hr) is now the preferred option of many civil society and public sector organisation to reduce exposure of children to HFSS food advertisings[15 16] .[17] .[18]

Study aims

The consultations on the Ofcom regulations on the restriction of television food advertising to children offers an opportunity to analyse responses from a range of stakeholder groups to a consultation on an important policy that aims to promote dietary public health through regulation of the food industry. We aimed to identify which arguments, and from which stakeholder groups, appeared to be most influential in shaping the changes in Ofcom's position from the initial consultation to the final recommendations.

Methods

We followed the Standards for Reporting Qualitative Research[19] in reporting our findings.

Patient and Public Involvement

This study did not involve use of patient identifiable data and only used publicly-available responses from stakeholder groups. We did not consult the public on the methods.

Data Sources

We qualitatively analysed all written responses from stakeholder groups to the 2006-7 Ofcom public consultation on the regulation of television advertising of food and drink to children. The consultation asked for responses to a series of questions regarding the various policy packages outlined by Ofcom. Options such as having a 9pm watershed before which HFSS foods could not be advertised, self-regulation, having a transitional period and exemptions to the regulations were asked about. Responses were freely available on the Ofcom website[20] and responses to both the first and second consultations were included. Responses from individual members of the public were not included as they tended to be very brief and non-specific. We therefore focused our analysis on key stakeholder organisations representing key constituencies. Where needed, Optical Character

For peer review only - http://bmjopen.bmj.com/site/about/guidelines.xhtml

136 Recognition software was used to transcribe the responses. The consultation questions can
137 be seen in Table A in the Appendix.

138

139 ***Data Analysis***

140 Conventional thematic content analysis[21] was used to analyse the data and the Framework
141 method[22] used to organise and chart data. This method involves creating coding categories
142 directly from the data and organising coding within a flexible matrix, which can then be
143 adjusted as more codes emerge from the text. As existing literature on the topic of
144 stakeholder influence on public health policy is limited, rather than using preconceived
145 categories with which to code the data, a new framework for analysis was developed, based
146 on no *a priori* assumptions. After familiarisation with the data, coding was performed line by
147 line for each of the responses from interested parties in NVivo (software developed by QSR
148 International for qualitative research).

149 Each response was assigned to a category based on the person or organisation from which it
150 originated to stratify responses between the various types of interested parties (Table 2).
151 These categories were initially determined by assigning labels to each response and then
152 subsequently refined by the reviewers. A list of each group classified by category can be
153 found in the Appendix Tables B1 and B2. The longest and second longest submissions from
154 each category were then coded to develop the initial framework.

155 Table 2: The categories into which stakeholder groups were classified.

**Category**	**Definition**
Advertising stakeholders	Advertising companies and representative bodies
Broadcast stakeholders	Broadcasting companies and representative bodies
Civil society groups	Groups that represent the interests of all or some of the general population. This does not include groups that may have

For peer review only

	affiliations with industry who would be included in one of the 'stakeholders' groups.
Food manufacturers	Companies that produce and sell food to retailers
Food retailers	A company that sells food to the general population
Food industry representative groups	Bodies that represent the interests of groups of food manufacturers and retailers
Politicians	Persons professionally involved in politics
Public health stakeholders	Groups that focus on promoting the health of the population

For peer review only

Following coding of the first two longest responses in each category by AR, a set of codes to apply to further responses was agreed between all authors. Codes were also grouped into themes at this stage to provide the most meaningful thematic coding of the data. The remaining responses were all coded using this analytical framework by AR with additional codes being created when needed. Once each of the responses was coded, a 10% sample of the data were independently duplicate-coded by one of the other authors (JA or MW) in order to ensure appropriate categorisation of the various codes and code hierarchy, and to improve internal validity. Using a matrix, the data were charted resulting in a summary of the data by category from each transcript. Illustrative quotations were highlighted at this point.

The resulting charted data were then interpreted and analysed to determine recurrent themes or topics. These were explored further using quotations to demonstrate the range of opinions in relation to each theme or topic. The positions taken by the interested parties were then compared to Ofcom's starting position and final statement, to identify which positions from which stakeholders appeared to have held the most influence on Ofcom's final position.

Ethics

Ethical permission was not sought for this study. The consultation responses used have been made freely available on the Ofcom website with the full knowledge of their authors. We, therefore, treat this as publically available data which does not require ethical permission for analysis. As we did not seek informed consent from the authors of consultation responses, we do not name them here – although names were provided on the Ofcom website. Instead, we have used only the categories described in Table 2 to identify quotations in our results. This also avoided the study from becoming too focused on specific stakeholders rather than building a general picture of arguments used by different stakeholder groups.

Results

Of 1136 responses received to both rounds of consultation, 997 were from individual members of the public (and thus excluded from the analysis); 139 were from stakeholder groups and were included in the analysis; 114 were responses to the initial consultation and 25 responses to the second consultation. The vast majority of responses from individuals were one-line statements of support for some form of restrictions without directly addressing specific issues concerning implementation. As such it was determined that there was not sufficient detail to determine arguments used, or positions taken. Therefore, these responses are unlikely to have influenced Ofcom other than to reaffirm that there was public support for some form of restriction.

The stakeholder responses varied in length from a few lines to double-digit numbers of pages. Most took the form of an initial broad statement outlining a policy position with supporting evidence, followed by shorter responses directed at addressing the specific questions in the consultation as outlined by Ofcom (shown in Table A, Appendix).

For peer review only - http://bmjopen.bmj.com/site/about/guidelines.xhtml

The organisations in the stakeholder groups outlined in Table 2 broadly fell into two separate categories. Civil society groups, politicians and public health stakeholders were encouraging of restrictions in order to reduce the exposure of children to advertising of HFSS foods. Advertising stakeholders, broadcast stakeholders, food manufacturers, food retailers and food industry stakeholders argued that restrictions would minimally impact childhood obesity whilst having a substantial impact on businesses. Though there were subtleties within each group with regards to what level of restrictions would be ideal, there were not sufficient differences in order to further analyse the differences in responses of the various stakeholder groups beyond these two broad categories.

The key changes from the initial Ofcom position to the final recommendations are summarised in Table 3. Arguments relating to each of the principles below, as outlined in the recommendations, were captured from the framework and are described in detail.

Table 3: The changes in Ofcom's position during the course of the consultation

**Initial options presented by Ofcom**	**Consultation responses and Ofcom's reaction**	**Ofcom's final position**	**Reference in consultation**
Ofcom's packages 1-3 varied on 3 key principles:			
1. Restrictions on advertising of all foods versus just HFSS foods	Following the first consultation it was clear that the majority of responses preferred restricting advertising of only HFSS foods.	The eventual package of restrictions enacted was specific to HFSS foods.	Ofcom Executive Summary 1.12
2. Total ban on food advertising versus volume-based restrictions	Almost all stakeholders did not consider volume-based restrictions as being effective at reducing exposure to advertising and this option	There was a total ban enacted on HFSS food advertising in programming 'of	Ofcom Executive Summary 1.12

For peer review only

	was dismissed following the first consultation.	particular interest to' children.	
3. Restrictions only on children's channels versus all programmes 'of particular interest' to children, irrespective of channel	Some responses highlighted that children may watch adult TV and a ban on all less healthy food advertising before a 9pm watershed may be more effective than focusing specifically on children's programming. Other responses worried that this would disproportionately impact advertising revenues.	Ofcom rejected the idea of a pre-9pm ban due to concerns about the effect it would have on broadcasters, programming and advertising revenues.	Ofcom Executive Summary 1.12
Further changes that were made:			
Restrictions should apply to children aged 4-9 years	Many responses pointed out that children are legally defined as under 16 years.	The restrictions applied to children aged 4-15 years.	Ofcom Final Statement 4.9
All restrictions should start in April 2007	Children's channels argued that they should be allowed a transitional period as they would be affected financially.	Children's channels were allowed a phased implementation of restrictions, with final implementation by January 2009.	Ofcom Final Statement 5.3/5.4

To which foods should restrictions apply?

For peer review only

There was non-partisan agreement that having a blanket ban on all television food advertising was counter-productive and had the possibility of inadvertently reducing exposure of children to advertisements for healthier products.

Quotes: Should restrictions apply to all foods?

"We do not support any options which would restrict advertising of all foods, including foods such as fruit and vegetables, milk and dairy products. These foods can play an important part in children consuming a balanced diet, and we consider that advertising can play a useful role in educating both parents and children in the ways to achieve this." (Food industry stakeholder)

"[Public health stakeholder] believes that it is desirable to distinguish between healthy and unhealthy foods. We do not believe it would be useful to restrict the advertising of all foods because this would mean manufacturers and retailers would be unable to promote healthy foods, such as fresh fruit and vegetables." (Public health stakeholder)

As the underlying aim of the restrictions was to protect health, preventing the advertising of healthy products would be counter-productive. Stakeholder groups agreed that banning advertisements of all foods would be deleterious to efforts to promote healthy eating and promoting a balanced diet.

Total ban or volume-based ban?

The idea of a broad volume-based restriction rather than a total ban targeting children's programming was proposed in Package 3 and was nearly universally disliked. Broadcasters, advertisers and food industry stakeholders argued that a volume-based restriction would have a very large effect on commercial revenues, whereas public health stakeholders and civil society groups cited how little a volume-based restriction would actually reduce the exposure of children to HFSS food advertising.

Quotes: Would a volume-based restriction be effective?

For peer review only - http://bmjopen.bmj.com/site/about/guidelines.xhtml

"The least acceptable option would be Package 3, which would have a devastating effect on our overall revenues - several times greater than Ofcom has estimated – while delivering a smaller reduction in the number of times children see food and drink adverts." (*Broadcast* stakeholder)

"Package 3 not only restricts the option to promote healthy foods to children, but also fails to restrict HFSS adverts during periods of viewing when many children are still watching i.e. up to 9pm." (Public health stakeholder)

Many responses argued that Package 3 would result in very little change in exposure of children to television advertising of HFSS foods but would substantially impact broadcasters and advertisers financially. Arguments concerning commercial impacts were used throughout the responses of industry groups, with emphasis on the fact that as a broadcast regulator, Ofcom has a duty to minimise impact on revenues for broadcasters.

Restrictions on children's programming or a pre-9pm watershed ban?

Although not included in any of Ofcom's proposals, one of the consultation questions asked about whether restricting advertising before 9pm would be a suitable measure. In response, civil society groups and public health stakeholders called for restricting all HFSS food advertising before a 9pm 'watershed'. Advertisers, broadcasters and the food industry claimed such restrictions would impinge upon adult viewing. All three groups highlighted the trade-off between protecting children and the loss of advertising exposure to adults. Advertisers, broadcasters and food industry groups cited the negative commercial impacts of a pre-9pm watershed ban as outweighing any 'marginal' public health benefits; whereas civil society groups and public health groups saw the public health benefit of a pre-9pm watershed ban as outweighing commercial impacts.

Quotes: Arguments pertaining to the pre-9pm watershed ban on HFSS food advertising

"[Food industry stakeholder organisation] welcomes Ofcom's rejection of the pre-9pm watershed, as this would have been tantamount to a complete ban on the advertising of

For peer review only

For peer review only - http://bmjopen.bmj.com/site/about/guidelines.xhtml

food and soft drink products on television, and would have impacted on adult airtime." (Food industry stakeholder)

"We believe that the most suitable option is the pre 9pm ban of HFSS advertising, for the following reasons:

- *achieves one of the key regulatory objectives, that of significantly reducing the impact of HFSS advertising on younger children*
- *removes 82% of the recorded HFSS advertising impacts on all children (aged 4-15)*
- *contributes substantially to enhancing protection for older children by reducing their exposure to HFSS advertising*
- *offers the greatest social and health benefits of all options – in the ranges of £50 million - £200 million per year or £250million - £990 million per year (depending on the value of life measure)".* (Civil society group)

"The avoidance of intrusive regulation of advertising during adult airtime is only justifiable once full account has been taken to address the over-riding priority to protect children's health. At times when adults and children are watching, the need to protect children must take priority." (Public health stakeholder)

In their final statement following the consultation,[23] Ofcom explained why they had rejected banning HFSS food advertising before a 9pm watershed due to the effect this was expected to have on adult viewing times and commercial revenues. Industry groups appeared to be successful in arguing that adult viewing should be unaffected despite the possibility that both children and adults may be watching television together. The need to protect the right of adults to see whatever they wish was a common argument against restricting advertising on television channels that were not explicitly targeted at children. The individual freedom of an adult therefore appeared to be given precedence over exposing children to HFSS food advertising.

Ofcom's research[23] showed that 48% of parents supported restricting HFSS food advertising before 9pm, which was often cited by industry responses as evidence of a lack of public support. Some responses highlighted the fact that the complete figures were 48% in support of a pre-9pm watershed ban, 24% against the ban, with the remainder undecided. An

For peer review only - http://bmjopen.bmj.com/site/about/guidelines.xhtml

apparently valid complaint made by public health groups regarding this issue was that Ofcom did not ever consult on a pre-9pm watershed ban despite its own research showing this would reduce the exposure of children to HFSS advertising by 82%.

We are also able to see here the use of evidence-based arguments by the civil society group in making their case. Some civil society groups and public health stakeholders would cite evidence to support their argument. The quotes above illustrate an example of how a civil society group used data and evidence to support their arguments by, for instance, suggesting that banning advertising prior to 9pm could reduce advertising exposure of children by 82%. This figure was taken from Ofcom's own analysis of the effects of the various policy options, which can now be found included in Ofcom's final report on the consultation.[23] Food industry representative groups on the other hand tended to cite a lack of evidence or only used evidence that appeared to support their arguments..

Quotes: Arguments regarding available evidence and its interpretation

"As Ofcom hasfound from its own research, television advertising has only a "modest direct effect" on children's food preferences, consumption and behaviour, and that other factors – including taste, price familiarity, peer pressure and convenience - all have a higher effect. Hastings, in his report for the Food Standards Agency, found that advertising had only a 2% direct effect on children's choice." (Food company)

"Ofcom quotes an estimate that advertising/television accounts for some 2% of variation in food choice/obesity. This is not a small figure considering that calculations by the Institute of Medicine show that this would mean an estimated additional 1.5 million young people in the US falling into the obese category." (Public health interests)

"The evidence that television has anything but an extremely small impact on the HFSS element of the diet of children is unconvincing and accordingly it is difficult to support proposals that appear disproportionate." (Broadcast interests)

To what ages of children should the restrictions apply?

Ofcom initially planned to restrict advertisements targeted at children aged 4-9 years, although this was subsequently expanded to cover children ages 4-15 years in the final

For peer review only

regulations. Children under 4 years were thought to have little influence over what foods and drinks were given to them and therefore not considered as part of the restrictions. Throughout the consultation food industry representative groups and food manufacturers argued that restricting advertisements to children aged 4-9 years was appropriate, whereas as public health stakeholders argued that this should be expanded to cover children aged 4-15 years.

Quotes: Arguments pertaining to the age of children to which restrictions should apply

"It is neither logical nor is there any explanation as to why Ofcom should propose to limit the focus of regulation to children aged under 10. The government asked Ofcom to consider proposals for strengthening its rules on television advertising of food to children. It did not ask Ofcom to limit its focus to any particular age group. Ofcom should logically apply restrictions according to its own definition of children (aged 15 [or under])." (Public health stakeholder)

"Children develop and refine their ability to interpret advertising messages as they get older. Existing studies suggest that by 10 years old (indeed, most studies suggest an even earlier age) they are considered to have sufficient cognitive development to understand the implications of television advertising." (Food manufacturer)

"We are alarmed by the decision to extend volume and scheduling restrictions of food and drink advertising to children under 16. The intention of Ofcom and the government has always been to protect younger children and industry responded on this basis. Ofcom has previously stated that it wished to find a proportionate solution and we question the evidence base on which this decision was made. A review of Ofcom's own literature would seem to contradict the question put to consultation and support the conclusion that young people are capable of differentiating between programming and advertising." (Food industry representative group)

For peer review only

For peer review only - http://bmjopen.bmj.com/site/about/guidelines.xhtml

The logic of defining children as aged 4-9 years was questioned by many stakeholders as, according to Ofcom and in the UK, children are legally defined as those under the age of 16 years. A number of food manufacturers stated that they already did not advertise their products to children under 8-12 years. They argued that during adolescence children become ‘media literate’ and are able to understand advertising and should therefore not be a target of the restrictions.

Industry arguments appeared to suggest that media ‘illiterate’ children need protecting from HFSS food advertising whereas public health groups suggested all children needed protecting regardless of how ‘media literate’ they are. Public health groups argued that adolescents are still susceptible to advertising, have more purchasing power and greater pester power than younger children, and may not appreciate the health implications of a poor diet. Ofcom concluded that expanding restrictions to include children aged 4-15 years was appropriate, suggesting the arguments of public health groups held more weight over this issue.

When should the restrictions start?

The need for a transitional period was also hotly debated. Public health stakeholders and civil society groups suggested that as companies were already aware that restrictions were due to be enforced any transitional period should be minimal. Industry groups argued that a transition period was necessary to allow adjustments to be made.

Quotes: Arguments pertaining to the need for a transitional period

“We do not believe [a] transitional period is appropriate. The arguments for “phasing in” restrictions appear to be of a commercial nature and not supportive of the policy’s public health objectives.” (Public health stakeholder)

“We would ask for a transitional period of at least three years. This would allow production companies to adjust, and the growing number of public companies to issue profit warnings where necessary.” (Broadcast stakeholder)

For peer review only - http://bmjopen.bmj.com/site/about/guidelines.xhtml

Instead of starting restrictions soon after announcement of the final policy statement (February 2007), a phased transition over 1-2 years was implemented (varying for different channel types), suggesting industry arguments held more weight on this point. Despite the stated objective of minimising the exposure of children to HFSS food advertising, it appears that Ofcom was more concerned about the potential commercial impact of advertising restrictions and delayed enforcement of the restrictions as a result.

For peer review only

Discussion

Summary of principal findings

This study presented a unique opportunity for a detailed analysis of responses to a public consultation on a public health policy in the UK. Such data is often not in the public domain and these data therefore offered a rare opportunity for scientific scrutiny. For example, verbatim responses to the 2016 consultation on the UK Soft Drinks Industry Levy, have not been released. Our paper highlights how, despite the relative transparency of the 2006-7 consultation, the final policy appeared to be substantially influenced by stakeholders. Commercial and public health interests aligned with regards to whether restrictions should apply to all foods or just HFSS foods as neither wished to ban advertising of healthy foods. Likewise, common ground was found when considering a volume-based ban, with it having large commercial impact but little public health impact as per Ofcom's own findings.[23]

As far as we are aware, this is the first analysis to examine how a range of stakeholder groups influenced the development of a public health policy aiming to regulate food industry advertising. Ofcom's decision to implement Modified Package 1 contained concessions to commercial as well as civil society and public health stakeholders. However, ultimately industry arguments appeared to hold more sway, with the main concession to public health groups being expanding restrictions from children aged 4-9 years to those aged 4 to 15 years. For the most part, Ofcom appeared to make concessions to industry arguments. Ofcom appeared to believe that the commercial impact of the regulation of advertising should carry greatest weight, even when the aim of the regulation was to protect children's health. As such, Ofcom did not formally consider a pre-9pm ban as part of any of its packages, as had been proposed by public health and civil society stakeholders, although one of the consultation questions did refer to a pre-9pm ban. Instead, Ofcom approved a two-year transition period and emphasised the need for 'proportionate action'. Some responses to the consultation from public health advocates argued that Ofcom, being a broadcast regulator rather than a public health stakeholder, felt an obligation to protect industry interests. The case for restricting advertising was made in a Department of Health 'white paper'[24] (NHS Strategy documents are known as 'white papers'). However, Ofcom was tasked with determining how to implement these restrictions. Under the

For peer review only - http://bmjopen.bmj.com/site/about/guidelines.xhtml

Communications Act 2003, Ofcom retains direct responsibility for advertising scheduling policy. This then begs the question of whether a governmental body with a duty to protect broadcasting interests should be leading on public health legislation.

This conflict between Ofcom's duties to the public and to broadcasters, may have resulted in eventual restrictions that did not appear to alter the level of exposure of children to HFSS food advertising.[13,14] Ofcom appeared to balance arguments related to commercial and public interests, in terms of jobs and the wider economy, with those relating to public health. Being proportionate in their restrictions was frequently cited by Ofcom in their decision making. Ofcom did not, however, appear to consider the cost to the economy of poor health that could stem from a lack of appropriate restrictions. Although this was cited by some public health groups (see quotes pertaining to a pre-9pm ban) this does not appear to have been considered by Ofcom in their final report, with no mention of wider societal costs. Ofcom also appeared to give greater priority to allowing advertisers access to adults than to restricting exposure to HFSS food advertising among children, who may be viewing the same programming. Industry representative groups tended to highlight commercial arguments whilst citing evidence that appeared to downplay the role of television advertising in childhood obesity. Public health groups emphasised that the health of children should outweigh any financial concerns and pointed out that even small changes to advertising at an individual level would affect large numbers of children and so accrue to large population level benefits.

Strengths and Limitations

Using established qualitative methods allowed us to identify key themes in the consultation responses according to stakeholder interests. The creation of a *de novo* framework minimised bias that might have been imposed by using a pre-existing framework. Instead, we allowed categories to emerge from the data. The classification of the responses also enabled us to see what positions were taken by the various stakeholders and which type of responses carried the most influence. Measures were taken to maximise the reliability of our coding, such as duplicate coding a sample of consultation responses. The use of publicly

For peer review only - http://bmjopen.bmj.com/site/about/guidelines.xhtml

available data was resource efficient. Additionally, the use of all the available data ensured that no perspectives were omitted, adding to internally validity. The omission of responses from individual members of the public was because most public responses lacked detail and were no more than a sentence long. Commercial influences on public health policy are unlikely to have changed over the past decade with no changes in lobbying rules or policy making procedures, making it highly likely that our findings from the 2007 consultation are applicable today.

There may be alternative methods by which the public influences policy making, such as by writing to their Member of Parliament. This is a study of only one case of public health policy making and our specific findings may not be generalisable to other aspects of dietary public health policy specifically or public health policy more generally. In this consultation, all members of a stakeholder category were treated as one, though there was some inter-category variation on position. There are also other ways by which interested parties could influence Ofcom, which we were unable to examine in this study. For example, Ofcom gave the option of providing confidential responses which were not available for us to incorporate into our dataset. Other informal lobbying may have occurred. Whether such channels of influence were used or whether similar arguments will have been used privately as were used publicly is unclear.

Relationship to existing knowledge

Some literature exists on the methods by which public health advocates influence policy. In 2006, the New Zealand government held an 'Inquiry into Obesity' in order to determine what could be done to limit increasing obesity rates.[12] Jenkin *et al* found that in three out of four domains examined, the governmental position aligned with that of industry groups, with the exception being nutritional policy in schools. In the other three domains, national obesity strategy, food industry policy, and advertising and marketing policy, the analysis determined that the governmental position allied with industry groups. Much like our study, public health groups were shown to have a limited impact on the eventual policies, with industry arguments proving more influential. An explanation suggested for this was the

For peer review only - http://bmjopen.bmj.com/site/about/guidelines.xhtml

significance of the food industry to New Zealand's economy, highlighting how considerations outside of public health may importantly shape public health policy. It may be the case that similar factors shaped the eventual restrictions in our case study, despite the appearance of Ofcom wanting to develop 'proportionate restrictions', balancing commercial and public health interests. The question of what is proportionate appears to be determined by ideology and how much one feels government's role is to protect health even if it impacts on industry. If this is the case, we must question whether commercial companies can ever be truly motivated to improve health at the possible detriment to their short-term profits. A thematic analysis of alcohol industry documents in Australia[25] concluded that the industry attempted to create an impression of social responsibility whilst promoting interventions that did not affect their profits and campaigning against effective interventions that might affect profits. The *de facto* exemption of commercial stakeholders from bearing the negative external costs of their profitable endeavours (e.g. environmental, social or health impacts) has been widely questioned.[26]

Interpretation and implications of the study

Much of the research undertaken to date on stakeholder influences on public health policy has focused on industry behaviours and practices, whereas in this study we have treated both pro-industry and pro-public health groups equally in our analysis. Our findings suggest that, in the case of the Ofcom consultation on the regulation of TV advertising of foods to children, civil society and public health stakeholders carried less weight than their industry counterparts. Industry groups were apparently successfully able to argue that extensive restrictions would impact upon their commercial revenues, suggesting that their economic arguments importantly influenced the thinking of policy-makers. However, the future (external) costs of treating the potential health implications of HFSS food consumption did not appear to influence policy-making. This may be because any potential cost-savings are long-term and would apply to the health sector, for which Ofcom has no governmental responsibility, whereas the short-term costs would apply to the broadcast sector for which Ofcom is the regulatory body.

For peer review only - http://bmjopen.bmj.com/site/about/guidelines.xhtml

Public health advocacy is an activity in which many public health professionals are keen to become more effective to better ensure that evidence is translated into policy.[27,28] This study highlights that responding to public health policy consultations alone may not result in policy making favourable to public health and other avenues of influence may also need to be explored. Conversely, the change in the definition of children from 4-9 years to 4-15 years demonstrates that there is scope for public health advocates to shape policy should an issue be sufficiently clear and difficult to oppose. A more Machiavellian interpretation would be that to define children as aged 4-9 years at the outset may have been a cynical ploy aimed at ensuring that there was at least some ground to concede to public health stakeholders and distract from the more contentious issues. This is supported by the fact that the definition of children as aged 4-9 years was inherently questionable, given Ofcom's own definition of children as under 16 years, in line with the legal and medical definitions used in the UK. A few companies pointed to their media literacy campaigns as evidence that adolescents can understand advertising as an argument against redefining the scope of these restrictions to children aged 4-15 years. Evidence shows that advertisers simply use different ways to target adolescents,[29] rendering media literacy moot,[30] and suggesting that restrictions are still needed to protect adolescents.

The issue of TV advertising of less healthy foods remains highly politically sensitive and at the top of the public health strategy agenda for obesity.[18] Many UK public health organisations have recently campaigned to ban television advertising of less healthy foods before 9pm (the so-called 9pm watershed).[16,17,31–34] Our analysis of the 2006-7 consultation offers specific insights that could be influential in this ongoing national debate, in the same way as such analyses of historical documents have influenced tobacco control efforts in recent years.[10,35] The Ofcom regulation of television advertising of less healthy foods to children is one of few national public health policies of this sort to have been independently evaluated.[14,36] The independent evaluation found that the introduction of the regulations were not associated with a decrease in children's exposure to less healthy food advertising.[36] Our analysis sheds further light on why and how a regulatory policy that appears to have been ineffective in reducing children's exposure to less healthy food advertising came about. Publishing responses to public consultations in full is a key

For peer review only - http://bmjopen.bmj.com/site/about/guidelines.xhtml

component of transparent policy making. The UK Treasury's reluctance to make available responses to the Soft Drinks Industry Levy consultation is contrary to this principle.

Further questions and future research

How policy making is influenced through means other than public consultations should be further studied. Other means of applying political pressure such as political lobbying and having indirect relationships with positions of power are much more opaque and difficult to monitor. Parliamentary records of lobbying activity, copies of internal or leaked documents and registers of MPs interests may all be potential sources of data to explore these issues further. Interviews with former or current employees of policy forming bodies such as Ofcom, as well as other stakeholder groups could also be fruitful. Claims made during this consultation, such as industry claims of needing to issue profit warnings as a consequence of lost revenue from these restrictions, could be analysed. Thematic analysis of further documents such as the responses analysed in this study could provide valuable insight into whether a similar combination of commercial arguments and questioning scientific data is used across different public health policy consultations.

Conclusion

This analysis increases our understanding of how influential some stakeholders are in policy making and provides a framework from which further understanding of the influences on public health policy can be determined. From this case study, we can see that commercial influences on dietary public health policy-making appear to be somewhat greater than the influence of public health stakeholders and this imbalance may have contributed to the ultimately compromised legislation. In this case, the potential for commercial impacts of legislation promoting public health appeared to outweigh the anticipated population health benefits in policy decision making.

Authors' contributions – The authors declare that they have no competing interests. Responses were coded by AR with a sub-sample independently duplicate coded by JA or MW. AR, JA and MW contributed to the manuscript in terms of both writing and editing.

For peer review only - http://bmjopen.bmj.com/site/about/guidelines.xhtml

Licensing Statement

The Submitting Author has the right to grant and does grant on behalf of all authors of the Work (as defined in the below author licence), an exclusive licence and/or a non-exclusive licence for contributions from authors who are: i) UK Crown employees; ii) where BMJ has agreed a CC-BY licence shall apply, and/or iii) in accordance with the terms applicable for US Federal Government officers or employees acting as part of their official duties; on a worldwide, perpetual, irrevocable, royalty-free basis to BMJ Publishing Group Ltd ("BMJ") its licensees and where the relevant Journal is co-owned by BMJ to the co-owners of the Journal, to publish the Work in BMJ Open and any other BMJ products and to exploit all rights, as set out in our licence.

The Submitting Author accepts and understands that any supply made under these terms is made by BMJ to the Submitting Author unless you are acting as an employee on behalf of your employer or a postgraduate student of an affiliated institution which is paying any applicable article publishing charge ("APC") for Open Access articles. Where the Submitting Author wishes to make the Work available on an Open Access basis (and intends to pay the relevant APC), the terms of reuse of such Open Access shall be governed by a Creative Commons licence – details of these licences and which Creative Commons licence will apply to this Work are set out in our licence referred to above.

For peer review only

References

1. Global Health Observatory. WHO | Obesity. WHO.

2. WHO | Reducing the impact of marketing of foods and non-alcoholic beverages on children. *WHO*. 2014. http://www.who.int/elena/titles/guidance_summaries/food_marketing_children/en/. Accessed August 8, 2017.

3. Kraak VI, Vandevijvere S, Sacks G, et al. Policy & practice Progress achieved in restricting the marketing of high-fat, sugary and salty food and beverage products to children. *Bull World Heal Organ*. 2016;94:540-548. doi:10.2471/BLT.15.158667.

4. Raine KD, Lobstein T, Landon J, et al. Restricting marketing to children: Consensus on policy interventions to address obesity. *J Public Health Policy*. 2013;34(2):239-253. doi:10.1057/jphp.2013.9.

5. Harris JL, Pomeranz JL, Lobstein T, Brownell KD. A Crisis in the Marketplace: How Food Marketing Contributes to Childhood Obesity and What Can Be Done. *Annu Rev Public Health*. 2009;30(1):211-225. doi:10.1146/annurev.publhealth.031308.100304.

6. Cairns G, Angus K, Hastings G, Caraher M. Systematic reviews of the evidence on the nature, extent and effects of food marketing to children. A retrospective summary. *Appetite*. 2013;62:209-215. doi:10.1016/j.appet.2012.04.017.

7. WHO | Protecting children from the harmful effects of food and drink marketing. *WHO*. 2014. http://www.who.int/features/2014/uk-food-drink-marketing/en/. Accessed June 28, 2017.

8. Sibbald B. Sugar industry sour on WHO report. *CMAJ*. 2003;168(12):1585. http://www.pubmedcentral.nih.gov/articlerender.fcgi?artid=156706&tool=pmcentrez&rendertype=abstract. Accessed December 23, 2015.

9. Savell E, Fooks G, Gilmore AB. How does the alcohol industry attempt to influence marketing regulations? A systematic review. *Addiction*. 2016;111(1):18-32. doi:10.1111/add.13048.

10. Savell E, Gilmore AB, Fooks G, Weishaar H, Gilmore A. How Does the Tobacco Industry Attempt to Influence Marketing Regulations? A Systematic Review. Derrick

For peer review only

GE, ed. *PLoS One*. 2014;9(2):e87389. doi:10.1371/journal.pone.0087389.

11. Grüning T, Gilmore AB, McKee M. Tobacco industry influence on science and scientists in Germany. *Am J Public Health*. 2006;96(1):20-32. doi:10.2105/AJPH.2004.061507.

12. Jenkin G, Signal L, Thomson G. Nutrition policy in whose interests? A New Zealand case study. *Public Health Nutr*. 2012;15(8):1483-1488. doi:10.1017/S1368980011003028.

13. Adams J, Tyrrell R, Adamson AJ, White M. Effect of restrictions on television food advertising to children on exposure to advertisements for "less healthy" foods: repeat cross-sectional study. *PLoS One*. 2012;7(2):e31578. doi:10.1371/journal.pone.0031578.

14. Adams J, Hennessy-Priest K, Ingimarsdóttir S, Sheeshka J, Ostbye T, White M. Food advertising during children's television in Canada and the UK. *Arch Dis Child*. 2009;94(9):658-662. doi:10.1136/adc.2008.151019.

15. Children's Food Campaign. *Through the Looking Glass: A Review of Topsy-Turvy Junk Food Marketing Regulations*.; 2013. https://www.sustainweb.org/publications/through_the_looking_glass/. Accessed October 10, 2017.

16. Sugar Reduction The evidence for action. 2015. www.gov.uk/phe. Accessed October 10, 2017.

17. The Labour Party. Labour Manifesto. http://www.labour.org.uk/index.php/manifesto2017. Published 2017. Accessed June 28, 2017.

18. *Childhood Obesity: A Plan for Action Chapter 2 2 DH ID Box Title: Childhood Obesity: A Plan for Action, Chapter 2*.; 2018. www.nationalarchives.gov.uk/doc/open-government-licence/. Accessed November 27, 2018.

19. O'Brien BC, Harris IB, Beckman TJ, Reed DA, Cook DA. Standards for reporting qualitative research: a synthesis of recommendations. *Acad Med*. 2014;89(9):1245-1251. doi:10.1097/ACM.0000000000000388.

For peer review only

20. Ofcom. *Television Advertising of Food and Drink Products to Children: Final Statement*.

21. Hsieh H-F, Shannon SE. Three approaches to qualitative content analysis. *Qual Health Res*. 2005;15(9):1277-1288. doi:10.1177/1049732305276687.

22. Gale NK, Heath G, Cameron E, Rashid S, Redwood S. Using the framework method for the analysis of qualitative data in multi-disciplinary health research. *BMC Med Res Methodol*. 2013;13:117. doi:10.1186/1471-2288-13-117.

23. Ofcom. Television Advertising of Food and Drink Products to Children - Ofcom. https://www.ofcom.org.uk/consultations-and-statements/category-2/foodads_new. Accessed August 8, 2017.

24. Department of Health. *Choosing Health: Making Healthy Choices Easier*.; 2004. https://webarchive.nationalarchives.gov.uk/20120509221645/http://www.dh.gov.uk/prod_consum_dh/groups/dh_digitalassets/@dh/@en/@ps/documents/digitalasset/dh_133489.pdf. Accessed March 12, 2019.

25. Miller PG, de Groot F, McKenzie S, Droste N. Vested interests in addiction research and policy. Alcohol industry use of social aspect public relations organizations against preventative health measures. *Addiction*. 2011;106(9):1560-1567. doi:10.1111/j.1360-0443.2011.03499.x.

26. Stuckler D, Nestle M. Big food, food systems, and global health. *PLoS Med*. 2012;9(6):e1001242. doi:10.1371/journal.pmed.1001242.

27. Dorfman L, Krasnow ID. Public Health and Media Advocacy. *Annu Rev Public Health*. 2014;35(1):293-306. doi:10.1146/annurev-publhealth-032013-182503.

28. Herrick C. The post-2015 landscape: vested interests, corporate social responsibility and public health advocacy. *Sociol Health Illn*. 2016;38(7):1026-1042. doi:10.1111/1467-9566.12424.

29. Livingstone S, Helsper EJ. Does Advertising Literacy Mediate the Effects of Advertising on Children? A Critical Examination of Two Linked Research Literatures in Relation to Obesity and Food Choice. *J Commun*. 2006;56(3):560-584. doi:10.1111/j.1460-2466.2006.00301.x.

For peer review only - http://bmjopen.bmj.com/site/about/guidelines.xhtml

30. Montgomery KC, Chester J. Interactive Food and Beverage Marketing: Targeting Adolescents in the Digital Age. *J Adolesc Heal*. 2009;45(3):S18-S29. doi:10.1016/j.jadohealth.2009.04.006.

31. Sustain / Children's Food Campaign. Junk Food Marketing. https://www.sustainweb.org/childrensfoodcampaign/junk_food_marketing/. Accessed November 1, 2017.

32. Cancer Research UK. Being overweight or obese could cause around 700,000 new UK cancers by 2035 | Cancer Research UK. http://www.cancerresearchuk.org/about-us/cancer-news/press-release/2016-01-07-being-overweight-or-obese-could-cause-around-700000-new-uk-cancers-by-2035. Accessed November 1, 2017.

33. Obesity Health Alliance. Tackling Childhood Obesity - 2017 Election Manifesto. http://obesityhealthalliance.org.uk/policy/. Accessed November 1, 2017.

34. Faculty of Public Health. Start Well, Live Better. http://www.fph.org.uk/start_well%2C_live_better_-_a_manifesto. Accessed November 1, 2017.

35. Fooks GJ, Gilmore AB. Corporate philanthropy, political influence, and health policy. *PLoS One*. 2013;8(11):e80864. doi:10.1371/journal.pone.0080864.

36. Adams J, Tyrrell R, Adamson AJ, White M. Effect of restrictions on television food advertising to children on exposure to advertisements for "less healthy" foods: repeat cross-sectional study. *PLoS One*. 2012;7(2):e31578. doi:10.1371/journal.pone.0031578.

For peer review only

For peer review only - http://bmjopen.bmj.com/site/about/guidelines.xhtml

Figure titles and legends

Figure 1: A timeline of the Ofcom process on developing new recommendations for limiting television food advertising to children.

Figure 1 legend:

* ’Interested parties’ are stakeholder groups who may have been affected by the proposed changes, including advertising agencies, advocacy groups, broadcasters, charities, healthcare associations, politicians, the food industry and the general public.

Table 1: Packages of regulations proposed by Ofcom in the initial consultation (March 2006)

Table 1 legend:

* HFSS food = High, Fat, Sugar and Salt foods

+ ‘of particular appeal to children’ = when the proportion of people watching who are children is more than 120% of the proportion of children in the UK population[23]

Table 2: The categories into which stakeholder groups were classified. A list of each group classified by category can be found in the Appendix.

Table 3: The changes in Ofcom’s position during the course of the consultation

Appendix

Table A: The questions Ofcom asked as part of the consultation

Table B1: The classification of the responses by organisational category

Table B2: The classification of the responses by organisational category (continued)

For peer review only - http://bmjopen.bmj.com/site/about/guidelines.xhtml

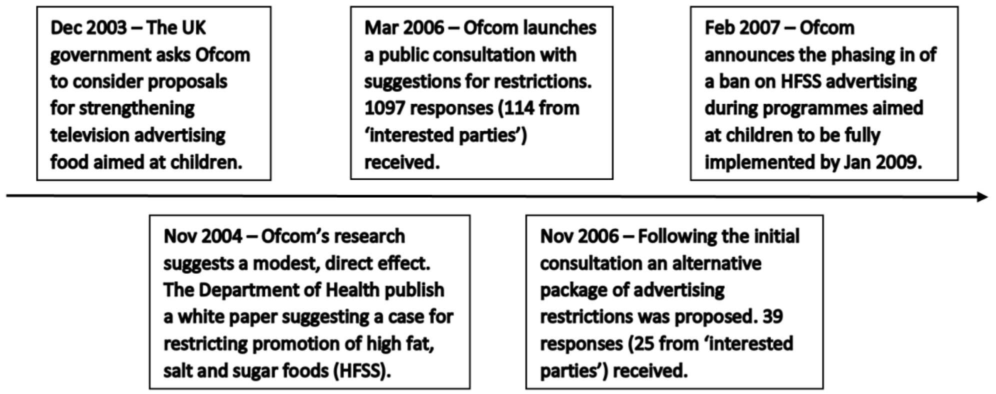

Figure 1: A timeline of the Ofcom process on developing new recommendations for limiting television food advertising to children.

Legend: * 'Interested parties' are stakeholder groups who may have been affected by the proposed changes, including advertising agencies, advocacy groups, broadcasters, charities, healthcare associations, politicians, the food industry and the general public.

Question 1	Do you agree that the regulatory objectives set out in paragraph 5.2 above are appropriate?
Question 2	Do you consider that it is desirable to distinguish between foods that are high in fat, salt or sugar and those that are healthier in order to achieve the regulatory objectives, or could an undifferentiated approach provide a reasonable alternative?
Question 3	If so, do you consider the FSA's nutrient profiling scheme to be a practical and reasonable basis for doing so? If not, what alternative would you propose? (Note: The nutrient profiling scheme was developed by the FSA and handed to Ofcom following extensive consultation (see FSA web site). This being the case, and given the scheme itself and the science upon which it is based fall outside Ofcom's area of responsibility and expertise, it is not appropriate in this consultation to seek responses on those matters)
Question 4	Do you agree that voluntary self-regulation would not be likely to meet Ofcom's regulatory objectives or the public policy objectives?
Question 5	Do you agree that the exclusion of all HFSS advertising before 9.00pm would be disproportionate?
Question 6	Do you agree that all food and drink advertising and sponsorship should be excluded from programmes aimed at pre-school children?
Question 7	Do you agree that revised content standards should apply to the advertising or sponsorship of all food and drink advertisements?
Question 8	Do you consider that the proposed age bands used in those rules aimed at preventing targeting of specific groups of children are appropriate?
Question 9	Do you consider the proposed content standards including their proposed wording to be appropriate, and if not, what changes would you propose, and why?
Question 10	Do you consider a transitional period would be appropriate for children's channels in the context of the scheduling restrictions, and if so, what measure of the 'amount' of advertising should be used?
Question 11	Do you consider there is a case for exempting low child audience satellite and cable channels from the provisions of Package 3?
Question 12	Do you agree that there should not be a phase-in period for children's channels under Package 3?
Question 13	Which of the three policy packages would you prefer to be incorporated into the advertising code and for what reasons?
Question 14	Alternatively, do you consider that a combination of different elements of the three packages would be suitable? If so, which elements would you favour within an alternative package? (You should note that the analysis in the Impact Assessment has focused on estimating the costs of restricting scheduling, volume, and content separately and would therefore allow consideration of other combinations of the same elements).
Question 15	Where you favour either Package 1 or 2, do you agree that it would be appropriate to allow children's channels a transitional period to phase in restrictions on HFSS / food advertising, on the lines proposed?
Question 16	Do you consider that the packages should include restrictions on brand advertising and sponsorship? If so, what criteria would be most appropriate to define a relevant brand? If not, do you see any issue with the prospect of food manufacturers substituting brand advertising and sponsorship for product promotion?
Question 17	Ofcom invites comments on the implementation approach set out in paragraph 5.45 and 5.46.

Advertising stakeholders	Broadcast stakeholders	Lawyers	Politicians	Retailers
Institute of Practitioners in Advertising	Producer's Alliance for Cinema and Television 1	Baker and McKenzie LLP	Mary Creagh MP	Sainsbury
Advertising Association	Five		Welsh Assembly	The Co-operative
Incorporated Society of British Advertisers 1	Channel 4		All Party Parlimentary group on Heart Disease	British Retail Consortium 1
Mediavest Manchester	Flextech television		David Amess MP	British Retail Consortium 2
Zenith Optimedia	ITV			
Mindshare	GMTV			
Incorporated Society of British Advertisers 2	Jetix, Nickelodeon and Turner			
	Producer's Alliance for Cinema and Television 2			
	Broadcast Advertising Clearance Centre			
	British Academy of Film and Television Arts			
	Broadcast Committee of Advertising Practice			

For peer review only - http://bmjopen.bmj.com/site/about/guidelines.xhtml

Food companies	Food industry representative groups	Civil society groups	Public health stakeholders
Kellogg 1	Food Advertising Unit 1	Kids Inc	National Heart Forum 1
Kraft 1	Food and Drink Federation 2	Consumer Council of Northern Ireland	Northern Ireland Chest Heart and Stroke 2
Pepsico	Snack Nut and Crisp Manufacturers Association 1	Children’s Food Bill Coalition	UK Public Health Association
GlaxoSmithKline 1	Food Advertising Unit 2	National Consumer Council 1	Royal Society of Health
United Biscuits	Food and Drink Federation 1	Which 1	Consensus Action on Salt and Health
Nestle	Dairy Council	Officer of the Children’s Commissioner	Nutrition Society
Cadbury	Snack Nut and Crisp Manufacturers Association 2	School Food Trust 1	Diabetes UK
Ferrero 1	Provision Trade Federation	Trading Standards Institute	Association for the Study of Obesity
Masterfoods 2	British Cheese Board	Which 2	Heart of Mersey 2
Coca-cola 1	Biscuit Cake Chocolate Confectionary Association 1	Welsh Consumer Council	National Oral Health Promotion Group
McDonalds 2	Biscuit Cake Chocolate Confectionary Association 2	Food Aware	Scientific Advisory Committee on Nutrition
Vimto	Dairy UK	Safefood Ireland	British Psychological Society
Wrigley		The Caroline Walker Trust	British Dietetic Association
Wiltshire farm foods		Advisory Committee for England	National Heart Forum 2
Unilever		Voice of the Listener and Viewer 2	British Heart Foundation
GlaxoSmithKline 2		Advertising Advisory Committess	British Medical Association 1
Coca-cola 2		British Nutrition Foundation	Cheshire and Merseyside Public Health Network
Masterfoods 2		Food Ethics Council	Health Protection Agency Northern Ireland
Kraft 2		Voice of the Listener and the Viewer 1	Irish Heart Foundation 1
McDonalds 1		National Consumer Council 2	National Heart Alliance Ireland 1
RHM Group		National Family and Parenting Institute	National Heart Alliance Ireland 2
Kellogg 2		National Union of Teaachers	International Association for the Study of Obesity 1
Ferrero 2		The Nutrition Society	British Medical Association 2
		Children’s Food Campaign	Heart of Mersey 1
		Consumer Council	Northern Ireland Chest Heart and Stroke 1
		Barnardos	Irish Heart Foundation 2
		National Children’s Bureau	NHS Borders
		Public Voice	Medical Research Council 1
		School Food Trust	British Heart Foundation 2
		Scotland’s Commissioner for Young People	Cancer Research UK
		Food Standards Agency	Northern Ireland Chest Heart and Stroke 3
		National Youth Agency	International Association for the Study of Obesity 2
		Advisory Committee for Northern Ireland	Royal College of Physicians
		Food Commission 1	Weight Concern
		Women’s Institute 1	British Dental Association
		The Food Commission	Medical Research Council 2
		The Obesity Awareness and Solutions Trust	Joint statement by the British Heart Foundation, Cancer Research UK and Diabetes UK
		National Federation of Women’s Institutes 1	Royal College of Nursing
		National Federation of Women’s Institutes 2	

Reporting checklist for qualitative study.

Based on the SRQR guidelines.

Instructions to authors

Complete this checklist by entering the page numbers from your manuscript where readers will find each of the items listed below.

Your article may not currently address all the items on the checklist. Please modify your text to include the missing information. If you are certain that an item does not apply, please write "n/a" and provide a short explanation.

Upload your completed checklist as an extra file when you submit to a journal.

In your methods section, say that you used the SRQR reporting guidelines, and cite them as:

O'Brien BC, Harris IB, Beckman TJ, Reed DA, Cook DA. Standards for reporting qualitative research: a synthesis of recommendations. Acad Med. 2014;89(9):1245-1251.

		Reporting Item	Page Number
	#1	Concise description of the nature and topic of the study identifying the study as qualitative or indicating the approach (e.g. ethnography, grounded theory) or data collection methods (e.g. interview, focus group) is recommended	5
	#2	Summary of the key elements of the study using the abstract format of the intended publication; typically includes background, purpose, methods, results and conclusions	2
Problem formulation	#3	Description and signifcance of the problem / phenomenon studied: review of relevant theory and empirical work; problem statement	4
Purpose or research question	#4	Purpose of the study and specific objectives or questions	5
Qualitative approach and research paradigm	#5	Qualitative approach (e.g. ethnography, grounded theory, case study, phenomenolgy, narrative research) and	5

For peer review only

		guiding theory if appropriate; identifying the research paradigm (e.g. postpositivist, constructivist / interpretivist) is also recommended; rationale. The rationale should briefly discuss the justification for choosing that theory, approach, method or technique rather than other options available; the assumptions and limitations implicit in those choices and how those choices influence study conclusions and transferability. As appropriate the rationale for several items might be discussed together.	
Researcher characteristics and reflexivity	#6	Researchers' characteristics that may influence the research, including personal attributes, qualifications / experience, relationship with participants, assumptions and / or presuppositions; potential or actual interaction between researchers' characteristics and the research questions, approach, methods, results and / or transferability	1
Context	#7	Setting / site and salient contextual factors; rationale	4
Sampling strategy	#8	How and why research participants, documents, or events were selected; criteria for deciding when no further sampling was necessary (e.g. sampling saturation); rationale	5
Ethical issues pertaining to human subjects	#9	Documentation of approval by an appropriate ethics review board and participant consent, or explanation for lack thereof; other confidentiality and data security issues	8
Data collection methods	#10	Types of data collected; details of data collection procedures including (as appropriate) start and stop dates of data collection and analysis, iterative process, triangulation of sources / methods, and modification of procedures in response to evolving study findings; rationale	5
Data collection instruments and technologies	#11	Description of instruments (e.g. interview guides, questionnaires) and devices (e.g. audio recorders) used for data collection; if / how the instruments(s) changed over the course of the study	6
Units of study	#12	Number and relevant characteristics of participants, documents, or events included in the study; level of	8

For peer review only

		participation (could be reported in results)	
Data processing	#13	Methods for processing data prior to and during analysis, including transcription, data entry, data management and security, verification of data integrity, data coding, and anonymisation / deidentification of excerpts	6
Data analysis	#14	Process by which inferences, themes, etc. were identified and developed, including the researchers involved in data analysis; usually references a specific paradigm or approach; rationale	6
Techniques to enhance trustworthiness	#15	Techniques to enhance trustworthiness and credibility of data analysis (e.g. member checking, audit trail, triangulation); rationale	7
Syntheses and interpretation	#16	Main findings (e.g. interpretations, inferences, and themes); might include development of a theory or model, or integration with prior research or theory	8
Links to empirical data	#17	Evidence (e.g. quotes, field notes, text excerpts, photographs) to substantiate analytic findings	11
Intergration with prior work, implications, transferability and contribution(s) to the field	#18	Short summary of main findings; explanation of how findings and conclusions connect to, support, elaborate on, or challenge conclusions of earlier scholarship; discussion of scope of application / generalizability; identification of unique contributions(s) to scholarship in a discipline or field	17
Limitations	#19	Trustworthiness and limitations of findings	18
Conflicts of interest	#20	Potential sources of influence of perceived influence on study conduct and conclusions; how these were managed	1
Funding	#21	Sources of funding and other support; role of funders in data collection, interpretation and reporting	1

The SRQR checklist is distributed with permission of Wolters Kluwer © 2014 by the Association of American Medical Colleges. This checklist was completed on 26. November 2018 using http://www.goodreports.org/, a tool made by the EQUATOR Network in collaboration with Penelope.ai

For peer review only

BMJ Open

What arguments and from whom are most influential in shaping public health policy: Thematic content analysis of responses to a public consultation on the regulation of television food advertising to children in the UK

Journal:	*BMJ Open*
Manuscript ID	bmjopen-2018-028221.R2
Article Type:	Research
Date Submitted by the Author:	25-Jun-2019
Complete List of Authors:	Razavi, Ahmed; MRC Epidemiology Unit<br>Adams, J; University of Cambridge, Centre for Diet & Activity Research<br>White, Martin; Newcastle University, Institute of Health and Society; University of Cambridge, Centre for Diet & Activity Research
**Primary Subject Heading**:	Public health
Secondary Subject Heading:	Health policy, Public health, Qualitative research
Keywords:	PUBLIC HEALTH, QUALITATIVE RESEARCH, Health policy < HEALTH SERVICES ADMINISTRATION & MANAGEMENT

SCHOLARONE™
Manuscripts

TITLE PAGE

Article Title: What arguments and from whom are most influential in shaping public health policy: Thematic content analysis of responses to a public consultation on the regulation of television food advertising to children in the UK

Authors:

Ahmed Razavi[1] (Academic Clinical Fellow) ahmed.razavi@nhs.net, Jean Adams[1] (Senior University Lecturer) jma79@medschl.cam.ac.uk, Martin White (Director of Research[1] and Visiting Professor[2]) martin.white@mrc-epid.cam.ac.uk

1. Centre for Diet & Activity Research, MRC Epidemiology Unit, University of Cambridge, School of Clinical Medicine, Cambridge, CB2 0QQ.
2. Institute of Health & Society, University of Newcastle upon Tyne, Newcastle NE2 4AX.

Corresponding author: Ahmed Razavi – ahmed.razavi@nhs.net

Key words: public health; health policy; diet; qualitative research; influence

4570 words

Competing interests - None

Funding - This work was undertaken by the Centre for Diet and Activity Research (CEDAR), a UKCRC Public Health Research Centre of Excellence. Funding from Cancer Research UK, the British Heart Foundation, the Economic and Social Research Council, the Medical Research Council, the National Institute for Health Research, and the Wellcome Trust, under the auspices of the UK Clinical Research Collaboration, is gratefully acknowledged. The funders had no role in the study design, data collection, analysis, interpretation, or writing, nor in the decision to submit the article for publication.

There was no patient or public involvement in the study design or conduct of the study.

Data availability - Data used was freely available from the Ofcom website until a recent update to the website following which data was removed from the website. Ofcom may be able to provide access to the responses on request.

For peer review only

Abstract

Objectives: We explore one aspect of the decision-making process - public consultation on policy proposals by a national regulatory body - aiming to understand how public health policy development is influenced by different stakeholders.

Design: We use thematic content analysis to explore responses to a national consultation on the regulation of television advertising of foods high in fat, salt and sugar aimed at children.

Setting: United Kingdom.

Results: 139 responses from key stakeholder groups were analysed to determine how they influenced the regulator's initial proposals for advertising restrictions. The regulator's priorities were questioned throughout the consultation process by public health stakeholders. The eventual restrictions implemented were less strict in many ways than those originally proposed. These changes appeared to be influenced most by commercial, rather than public health, stakeholders.

Conclusions: Public health policy-making appears to be considered as a balance between commercial and public health interests. Tactics such as the questioning and reframing of scientific evidence may be used. In this example exploring the development of policy regulating television food advertising to children, commercial considerations appear to have led to a watering down of initial regulatory proposals, with proposed packages not including the measure public health advocates considered to be the most effective. This seems likely to have compromised the ultimate public health effectiveness of the regulations eventually implemented.

Article Summary – Strengths and limitations of this study

- Established qualitative methodology (thematic content analysis) was used to evaluate all stakeholder responses.
- A *de novo* analytical framework was created, minimising bias that may have occurred from using a pre-existing framework.
- Stakeholder groups were sorted into eight broad categories allowing us to compare and contrast responses by category.

For peer review only - http://bmjopen.bmj.com/site/about/guidelines.xhtml

- Policy-making can be influenced through other non-public means (e.g. direct lobbying), making us unable to comment on how other methods of influencing policy-making may have affected this consultation's outcome.
- This is one case study of influencing policy and our findings may not be generalisable to other cases.

For peer review only

For peer review only - http://bmjopen.bmj.com/site/about/guidelines.xhtml

Background

Foods high in fat, salt and sugar (HFSS) are a contributing factor to increasing rates of non-communicable disease worldwide[1] and the World Health Organization (WHO) has encouraged member states to take action on non-communicable diseases, including through regulation of the advertising of HFSS foods.[2] However, a 2016 study found that no member states had implemented comprehensive legislation restricting marketing of unhealthy food and beverages to young people,[3] despite multiple systematic reviews demonstrating the importance of food marketing as a driver of childhood obesity.[4–6]

Industry groups often seek to influence public health policy.[7] For example, in 2003 a WHO recommendation suggesting reduction in population sugar intake resulted in the Sugar Association (a sugar industry information group) pressing the US Congress to cut WHO funding.[8] However, influences on dietary public health policy are not limited to the food industry. Health professionals, charities, politicians and members of the public have all attempted to influence policy making. Evidence of the impact of these activities is hard to find in peer-reviewed literature.

Systematic reviews [9,10,11] have demonstrated how the alcohol and tobacco industries focus on lobbying efforts and promote self-regulation as means to minimise the impact of public health policy on commercial activities. These tactics have also been seen in relation to food where, in one case study, government opinion reflected industry rather than public health opinion.[12] However, at present, we have limited insight into how stakeholders other than those representing industry interests attempt to influence public health policy in general or dietary public health policy in particular. Identifying strategies and arguments used by these interested parties in a public setting may help inform how public health policy is determined and how it might more effectively be developed in the future.

Policy context

In December 2003, the UK Government asked Ofcom (the UK communications industry regulator) to consider proposals for strengthening rules on television advertising of food aimed at children (Fig 1). Ofcom decided to use the Food Standards Agency's Nutrient Profiling Model to determine which foods were classified as HFSS. Ofcom originally put

For peer review only - http://bmjopen.bmj.com/site/about/guidelines.xhtml

three proposed ‘packages’ of regulations to public consultation in March 2006 (Packages 1-3 in Table 1). Following this, Ofcom produced an alternative package of restrictions (Modified Package 1 in Table 1) in November 2006, on which Ofcom again consulted.

Figure 1: A timeline of the Ofcom process on developing new recommendations for limiting television food advertising to children.

Table 1: Packages of regulations proposed by Ofcom in the initial consultation (March 2006)

**Options**	**Detail**
Package 1	• No HFSS[*] food advertising during programmes specifically made for children<br>• No HFSS food advertising during programmes of particular appeal to children[+] aged 4-9 years
Package 2	• No food or drink advertising during programmes made specifically for children or of particular appeal to children aged up to 9 years
Package 3	• Volume of food and drink advertising to be limited at times when children are most likely to be watching
Modified Package 1	• As per package 1 except restrictions on HFSS food advertising to be extended to programmes of particular appeal to children aged 4-15 years

* ’Interested parties’ are stakeholder groups who may have been affected by the proposed changes, including advertising agencies, advocacy groups, broadcasters, charities, healthcare associations, politicians, the food industry and the general public.

Following the second consultation (November 2006), modified package 1 was recommended by Ofcom and was implemented from January 2009. A comparison of the final regulations implemented to the initial packages proposed suggests that the consultations had substantial impacts on policy decisions. The only independent evaluation of the regulations eventually implemented found no change in the proportion of advertisements seen by children that were for HFSS foods from before to after

For peer review only

implementation and an increase exposure of HFSS advertising among adults.[13,14] A '9pm watershed' (i.e. no advertising of HFSS foods before 21.00hr) is now the preferred option of many civil society and public sector organisation to reduce exposure of children to HFSS food advertisings[15 16] .[17] .[18]

Study aims

The consultations on the Ofcom regulations on the restriction of television food advertising to children offers an opportunity to analyse responses from a range of stakeholder groups to a consultation on an important policy that aims to promote dietary public health through regulation of the food industry. We aimed to identify which arguments, and from which stakeholder groups, appeared to be most influential in shaping the changes in Ofcom's position from the initial consultation to the final recommendations.

Methods

We followed the Standards for Reporting Qualitative Research[19] in reporting our findings.

Patient and Public Involvement

This study did not involve use of patient identifiable data and only used publicly-available responses from stakeholder groups. We did not consult the public on the methods.

Data Sources

We qualitatively analysed all written responses from stakeholder groups to the 2006-7 Ofcom public consultation on the regulation of television advertising of food and drink to children. The consultation asked for responses to a series of questions regarding the various policy packages outlined by Ofcom. Options such as having a 9pm watershed before which HFSS foods could not be advertised, self-regulation, having a transitional period and exemptions to the regulations were asked about. Responses were freely available on the Ofcom website[20] and responses to both the first and second consultations were included. Responses from individual members of the public were not included as they tended to be very brief and non-specific. We therefore focused our analysis on key stakeholder organisations representing key constituencies. Where needed, Optical Character

For peer review only - http://bmjopen.bmj.com/site/about/guidelines.xhtml

Recognition software was used to transcribe the responses. The consultation questions can be seen in Table A in the Appendix.

Data Analysis

Conventional thematic content analysis[21] was used to analyse the data and the Framework method[22] used to organise and chart data. This method involves creating coding categories directly from the data and organising coding within a flexible matrix, which can then be adjusted as more codes emerge from the text. As existing literature on the topic of stakeholder influence on public health policy is limited, rather than using preconceived categories with which to code the data, a new framework for analysis was developed, based on no *a priori* assumptions. After familiarisation with the data, coding was performed line by line for each of the responses from interested parties in NVivo (software developed by QSR International for qualitative research).

Each response was assigned to a category based on the person or organisation from which it originated to stratify responses between the various types of interested parties (Table 2). These categories were initially determined by assigning labels to each response and then subsequently refined by the reviewers. A list of each group classified by category can be found in the Appendix Tables B1 and B2. The longest and second longest submissions from each category were then coded to develop the initial framework.

Table 2: The categories into which stakeholder groups were classified.

Category	Definition
Advertising stakeholders	Advertising companies and representative bodies
Broadcast stakeholders	Broadcasting companies and representative bodies
Civil society groups	Groups that represent the interests of all or some of the general population. This does not include groups that may have

For peer review only - http://bmjopen.bmj.com/site/about/guidelines.xhtml

	affiliations with industry who would be included in one of the 'stakeholders' groups.
Food manufacturers	Companies that produce and sell food to retailers
Food retailers	A company that sells food to the general population
Food industry representative groups	Bodies that represent the interests of groups of food manufacturers and retailers
Politicians	Persons professionally involved in politics
Public health stakeholders	Groups that focus on promoting the health of the population

Following coding of the first two longest responses in each category by AR, a set of codes to apply to further responses was agreed between all authors. Codes were also grouped into themes at this stage to provide the most meaningful thematic coding of the data. The remaining responses were all coded using this analytical framework by AR with additional codes being created when needed. Once each of the responses was coded, a 10% sample of the data were independently duplicate-coded by one of the other authors (JA or MW) in order to ensure appropriate categorisation of the various codes and code hierarchy, and to improve internal validity. Using a matrix, the data were charted resulting in a summary of the data by category from each transcript. Illustrative quotations were highlighted at this point.

The resulting charted data were then interpreted and analysed to determine recurrent themes or topics. These were explored further using quotations to demonstrate the range of opinions in relation to each theme or topic. The positions taken by the interested parties were then compared to Ofcom's starting position and final statement, to identify which positions from which stakeholders appeared to have held the most influence on Ofcom's final position.

For peer review only

For peer review only - http://bmjopen.bmj.com/site/about/guidelines.xhtml

Ethics

Ethical permission was not sought for this study. The consultation responses used have been made freely available on the Ofcom website with the full knowledge of their authors. We, therefore, treat this as publically available data which does not require ethical permission for analysis. As we did not seek informed consent from the authors of consultation responses, we do not name them here – although names were provided on the Ofcom website. Instead, we have used only the categories described in Table 2 to identify quotations in our results. This also avoided the study from becoming too focused on specific stakeholders rather than building a general picture of arguments used by different stakeholder groups.

Results

Of 1136 responses received to both rounds of consultation, 997 were from individual members of the public (and thus excluded from the analysis); 139 were from stakeholder groups and were included in the analysis; 114 were responses to the initial consultation and 25 responses to the second consultation. The vast majority of responses from individuals were one-line statements of support for some form of restrictions without directly addressing specific issues concerning implementation. As such it was determined that there was not sufficient detail to determine arguments used, or positions taken. Therefore, these responses are unlikely to have influenced Ofcom other than to reaffirm that there was public support for some form of restriction.

The stakeholder responses varied in length from a few lines to double-digit numbers of pages. Most took the form of an initial broad statement outlining a policy position with supporting evidence, followed by shorter responses directed at addressing the specific questions in the consultation as outlined by Ofcom (shown in Table A, Appendix).

For peer review only - http://bmjopen.bmj.com/site/about/guidelines.xhtml

The organisations in the stakeholder groups outlined in Table 2 broadly fell into two separate categories. Civil society groups, politicians and public health stakeholders were encouraging of restrictions in order to reduce the exposure of children to advertising of HFSS foods. Advertising stakeholders, broadcast stakeholders, food manufacturers, food retailers and food industry stakeholders argued that restrictions would minimally impact childhood obesity whilst having a substantial impact on businesses. Though there were subtleties within each group with regards to what level of restrictions would be ideal, there were not sufficient differences in order to further analyse the differences in responses of the various stakeholder groups beyond these two broad categories.

The key changes from the initial Ofcom position to the final recommendations are summarised in Table 3. Arguments relating to each of the principles below, as outlined in the recommendations, were captured from the framework and are described in detail.

Table 3: The changes in Ofcom's position during the course of the consultation

**Initial options presented by Ofcom**	**Consultation responses and Ofcom's reaction**	**Ofcom's final position**	**Reference in consultation**
Ofcom's packages 1-3 varied on 3 key principles:			
1. Restrictions on advertising of all foods versus just HFSS foods	Following the first consultation it was clear that the majority of responses preferred restricting advertising of only HFSS foods.	The eventual package of restrictions enacted was specific to HFSS foods.	Ofcom Executive Summary 1.12
2. Total ban on food advertising versus volume-based restrictions	Almost all stakeholders did not consider volume-based restrictions as being effective at reducing exposure to advertising and this option was	There was a total ban enacted on HFSS food advertising in programming 'of particular interest to' children.	Ofcom Executive Summary 1.12

For peer review only

	dismissed following the first consultation.		
3. Restrictions only on children's channels versus all programmes 'of particular interest' to children, irrespective of channel	Public health and civil society responses highlighted that children may watch adult TV and a ban on all less healthy food advertising before a 9pm watershed may be more effective than focusing specifically on children's programming. Television and advertising industry responses worried that this would disproportionately impact advertising revenues.	Ofcom rejected the idea of a pre-9pm ban due to concerns about the effect it would have on broadcasters, programming and advertising revenues.	Ofcom Executive Summary 1.12
Further changes that were made:			
Restrictions should apply to children aged 4-9 years	Many public health and civil society responses pointed out that children are legally defined as under 16 years.	The restrictions applied to children aged 4-15 years.	Ofcom Final Statement 4.9
All restrictions should start in April 2007	Children's channels argued that they should be allowed a transitional period as they would be affected financially.	Children's channels were allowed a phased implementation of restrictions, with final implementation by January 2009.	Ofcom Final Statement 5.3/5.4

217

218 ***To which foods should restrictions apply?***

For peer review only - http://bmjopen.bmj.com/site/about/guidelines.xhtml
For peer review only

There was non-partisan agreement that having a blanket ban on all television food advertising was counter-productive and had the possibility of inadvertently reducing exposure of children to advertisements for healthier products.

Quotes: Should restrictions apply to all foods?

"We do not support any options which would restrict advertising of all foods, including foods such as fruit and vegetables, milk and dairy products. These foods can play an important part in children consuming a balanced diet, and we consider that advertising can play a useful role in educating both parents and children in the ways to achieve this." (Food industry stakeholder)

"[Public health stakeholder] believes that it is desirable to distinguish between healthy and unhealthy foods. We do not believe it would be useful to restrict the advertising of all foods because this would mean manufacturers and retailers would be unable to promote healthy foods, such as fresh fruit and vegetables." (Public health stakeholder)

As the underlying aim of the restrictions was to protect health, preventing the advertising of healthy products would be counter-productive. Stakeholder groups agreed that banning advertisements of all foods would be deleterious to efforts to promote healthy eating and promoting a balanced diet.

Total ban or volume-based ban?

The idea of a broad volume-based restriction rather than a total ban targeting children's programming was proposed in Package 3 and was nearly universally disliked. Broadcasters, advertisers and food industry stakeholders argued that a volume-based restriction would have a very large effect on commercial revenues, whereas public health stakeholders and civil society groups cited how little a volume-based restriction would actually reduce the exposure of children to HFSS food advertising.

Quotes: Would a volume-based restriction be effective?

For peer review only - http://bmjopen.bmj.com/site/about/guidelines.xhtml

"The least acceptable option would be Package 3, which would have a devastating effect on our overall revenues - several times greater than Ofcom has estimated – while delivering a smaller reduction in the number of times children see food and drink adverts." (Broadcast stakeholder)

"Package 3 not only restricts the option to promote healthy foods to children, but also fails to restrict HFSS adverts during periods of viewing when many children are still watching i.e. up to 9pm." (Public health stakeholder)

Many responses argued that Package 3 would result in very little change in exposure of children to television advertising of HFSS foods but would substantially impact broadcasters and advertisers financially. Arguments concerning commercial impacts were used throughout the responses of industry groups, with emphasis on the fact that as a broadcast regulator, Ofcom has a duty to minimise impact on revenues for broadcasters.

Restrictions on children's programming or a pre-9pm watershed ban?

Although not included in any of Ofcom's proposals, one of the consultation questions asked about whether restricting advertising before 9pm would be a suitable measure. In response, civil society groups and public health stakeholders called for restricting all HFSS food advertising before a 9pm 'watershed'. Advertisers, broadcasters and the food industry claimed such restrictions would impinge upon adult viewing. All three groups highlighted the trade-off between protecting children and the loss of advertising exposure to adults. Advertisers, broadcasters and food industry groups cited the negative commercial impacts of a pre-9pm watershed ban as outweighing any 'marginal' public health benefits; whereas civil society groups and public health groups saw the public health benefit of a pre-9pm watershed ban as outweighing commercial impacts.

Quotes: Arguments pertaining to the pre-9pm watershed ban on HFSS food advertising

"[Food industry stakeholder organisation] welcomes Ofcom's rejection of the pre-9pm watershed, as this would have been tantamount to a complete ban on the advertising of

For peer review only - http://bmjopen.bmj.com/site/about/guidelines.xhtml

food and soft drink products on television, and would have impacted on adult airtime." (Food industry stakeholder)

"We believe that the most suitable option is the pre 9pm ban of HFSS advertising, for the following reasons:

- *achieves one of the key regulatory objectives, that of significantly reducing the impact of HFSS advertising on younger children*
- *removes 82% of the recorded HFSS advertising impacts on all children (aged 4-15)*
- *contributes substantially to enhancing protection for older children by reducing their exposure to HFSS advertising*
- *offers the greatest social and health benefits of all options – in the ranges of £50 million - £200 million per year or £250million - £990 million per year (depending on the value of life measure)".* (Civil society group)

"The avoidance of intrusive regulation of advertising during adult airtime is only justifiable once full account has been taken to address the over-riding priority to protect children's health. At times when adults and children are watching, the need to protect children must take priority." (Public health stakeholder)

In their final statement following the consultation,[23] Ofcom explained why they had rejected banning HFSS food advertising before a 9pm watershed due to the effect this was expected to have on adult viewing times and commercial revenues. Industry groups appeared to be successful in arguing that adult viewing should be unaffected despite the possibility that both children and adults may be watching television together. The need to protect the right of adults to see whatever they wish was a common argument against restricting advertising on television channels that were not explicitly targeted at children. The individual freedom of an adult therefore appeared to be given precedence over exposing children to HFSS food advertising.

Ofcom's research[23] showed that 48% of parents supported restricting HFSS food advertising before 9pm, which was often cited by industry responses as evidence of a lack of public support. Some responses highlighted the fact that the complete figures were 48% in support of a pre-9pm watershed ban, 24% against the ban, with the remainder undecided. An

For peer review only

apparently valid complaint made by public health groups regarding this issue was that Ofcom did not ever consult on a pre-9pm watershed ban despite its own research showing this would reduce the exposure of children to HFSS advertising by 82%.

We are also able to see here the use of evidence-based arguments by the civil society group in making their case. Some civil society groups and public health stakeholders would cite evidence to support their argument. The quotes above illustrate an example of how a civil society group used data and evidence to support their arguments by, for instance, suggesting that banning advertising prior to 9pm could reduce advertising exposure of children by 82%. This figure was taken from Ofcom's own analysis of the effects of the various policy options, which can now be found included in Ofcom's final report on the consultation.[23] Food industry representative groups on the other hand tended to cite a lack of evidence or only used evidence that appeared to support their arguments..

Quotes: Arguments regarding available evidence and its interpretation

"As Ofcom hasfound from its own research, television advertising has only a "modest direct effect" on children's food preferences, consumption and behaviour, and that other factors – including taste, price familiarity, peer pressure and convenience - all have a higher effect. Hastings, in his report for the Food Standards Agency, found that advertising had only a 2% direct effect on children's choice." (Food company)

"Ofcom quotes an estimate that advertising/television accounts for some 2% of variation in food choice/obesity. This is not a small figure considering that calculations by the Institute of Medicine show that this would mean an estimated additional 1.5 million young people in the US falling into the obese category." (Public health interests)

"The evidence that television has anything but an extremely small impact on the HFSS element of the diet of children is unconvincing and accordingly it is difficult to support proposals that appear disproportionate." (Broadcast interests)

To what ages of children should the restrictions apply?

Ofcom initially planned to restrict advertisements targeted at children aged 4-9 years, although this was subsequently expanded to cover children ages 4-15 years in the final

For peer review only

regulations. Children under 4 years were thought to have little influence over what foods and drinks were given to them and therefore not considered as part of the restrictions. Throughout the consultation food industry representative groups and food manufacturers argued that restricting advertisements to children aged 4-9 years was appropriate, whereas as public health stakeholders argued that this should be expanded to cover children aged 4-15 years.

Quotes: Arguments pertaining to the age of children to which restrictions should apply

"It is neither logical nor is there any explanation as to why Ofcom should propose to limit the focus of regulation to children aged under 10. The government asked Ofcom to consider proposals for strengthening its rules on television advertising of food to children. It did not ask Ofcom to limit its focus to any particular age group. Ofcom should logically apply restrictions according to its own definition of children (aged 15 [or under])." (Public health stakeholder)

"Children develop and refine their ability to interpret advertising messages as they get older. Existing studies suggest that by 10 years old (indeed, most studies suggest an even earlier age) they are considered to have sufficient cognitive development to understand the implications of television advertising." (Food manufacturer)

"We are alarmed by the decision to extend volume and scheduling restrictions of food and drink advertising to children under 16. The intention of Ofcom and the government has always been to protect younger children and industry responded on this basis. Ofcom has previously stated that it wished to find a proportionate solution and we question the evidence base on which this decision was made. A review of Ofcom's own literature would seem to contradict the question put to consultation and support the conclusion that young people are capable of differentiating between programming and advertising." (Food industry representative group)

For peer review only - http://bmjopen.bmj.com/site/about/guidelines.xhtml

The logic of defining children as aged 4-9 years was questioned by many stakeholders as, according to Ofcom and in the UK, children are legally defined as those under the age of 16 years. A number of food manufacturers stated that they already did not advertise their products to children under 8-12 years. They argued that during adolescence children become 'media literate' and are able to understand advertising and should therefore not be a target of the restrictions.

Industry arguments appeared to suggest that media 'illiterate' children need protecting from HFSS food advertising whereas public health groups suggested all children needed protecting regardless of how 'media literate' they are. Public health groups argued that adolescents are still susceptible to advertising, have more purchasing power and greater pester power than younger children, and may not appreciate the health implications of a poor diet. Ofcom concluded that expanding restrictions to include children aged 4-15 years was appropriate, suggesting the arguments of public health groups held more weight over this issue.

For peer review only

When should the restrictions start?

The need for a transitional period was also hotly debated. Public health stakeholders and civil society groups suggested that as companies were already aware that restrictions were due to be enforced any transitional period should be minimal. Industry groups argued that a transition period was necessary to allow adjustments to be made.

Quotes: Arguments pertaining to the need for a transitional period

"We do not believe [a] transitional period is appropriate. The arguments for "phasing in" restrictions appear to be of a commercial nature and not supportive of the policy's public health objectives." (Public health stakeholder)

"We would ask for a transitional period of at least three years. This would allow production companies to adjust, and the growing number of public companies to issue profit warnings where necessary." (Broadcast stakeholder)

For peer review only - http://bmjopen.bmj.com/site/about/guidelines.xhtml

Instead of starting restrictions soon after announcement of the final policy statement (February 2007), a phased transition over 1-2 years was implemented (varying for different channel types), suggesting industry arguments held more weight on this point. Despite the stated objective of minimising the exposure of children to HFSS food advertising, it appears that Ofcom was more concerned about the potential commercial impact of advertising restrictions and delayed enforcement of the restrictions as a result.

For peer review only

Discussion

Summary of principal findings

This study presented a unique opportunity for a detailed analysis of responses to a public consultation on a public health policy in the UK. Such data is often not in the public domain and these data therefore offered a rare opportunity for scientific scrutiny. For example, verbatim responses to the 2016 consultation on the UK Soft Drinks Industry Levy, have not been released. Our paper highlights how, despite the relative transparency of the 2006-7 consultation, the final policy appeared to be substantially influenced by stakeholders. Commercial and public health interests aligned with regards to whether restrictions should apply to all foods or just HFSS foods as neither wished to ban advertising of healthy foods. Likewise, common ground was found when considering a volume-based ban, with it having large commercial impact but little public health impact as per Ofcom's own findings.[23]

As far as we are aware, this is the first analysis to examine how a range of stakeholder groups influenced the development of a public health policy aiming to regulate food industry advertising. Ofcom's decision to implement Modified Package 1 contained concessions to commercial as well as civil society and public health stakeholders. However, ultimately industry arguments appeared to hold more sway, with the main concession to public health groups being expanding restrictions from children aged 4-9 years to those aged 4 to 15 years. Ofcom appeared to believe that the commercial impact of the regulation of advertising should carry greatest weight, even when the aim of the regulation was to protect children's health. As such, Ofcom did not formally consider a pre-9pm ban as part of any of its packages, as had been proposed by public health and civil society stakeholders, although one of the consultation questions did refer to a pre-9pm ban. Instead, Ofcom approved a two-year transition period and emphasised the need for 'proportionate action'. Some responses to the consultation from public health advocates argued that Ofcom, being a broadcast regulator rather than a public health stakeholder, felt an obligation to protect industry interests. The case for restricting advertising was made in a Department of Health 'white paper'[24] (NHS Strategy documents are known as 'white papers'). However, Ofcom was tasked with determining how to implement these restrictions. Under the Communications Act 2003, Ofcom retains direct responsibility for advertising scheduling

For peer review only - http://bmjopen.bmj.com/site/about/guidelines.xhtml

policy. This then begs the question of whether a governmental body with a duty to protect broadcasting interests should be leading on public health legislation.

This conflict between Ofcom's duties to the public and to broadcasters, may have resulted in eventual restrictions that did not appear to alter the level of exposure of children to HFSS food advertising.[13,14] Ofcom appeared to balance arguments related to commercial and public interests, in terms of jobs and the wider economy, with those relating to public health. Being proportionate in their restrictions was frequently cited by Ofcom in their decision making. Ofcom did not, however, appear to consider the cost to the economy of poor health that could stem from a lack of appropriate restrictions. Although this was cited by some public health groups (see quotes pertaining to a pre-9pm ban) this does not appear to have been considered by Ofcom in their final report, with no mention of wider societal costs. Ofcom also appeared to give greater priority to allowing advertisers access to adults than to restricting exposure to HFSS food advertising among children, who may be viewing the same programming. Industry representative groups tended to highlight commercial arguments whilst citing evidence that appeared to downplay the role of television advertising in childhood obesity. Public health groups emphasised that the health of children should outweigh any financial concerns and pointed out that even small changes to advertising at an individual level would affect large numbers of children and so accrue to large population level benefits.

Strengths and Limitations

Using established qualitative methods allowed us to identify key themes in the consultation responses according to stakeholder interests. The creation of a *de novo* framework minimised bias that might have been imposed by using a pre-existing framework. Instead, we allowed categories to emerge from the data. The classification of the responses also enabled us to see what positions were taken by the various stakeholders and which type of responses carried the most influence. Measures were taken to maximise the reliability of our coding, such as duplicate coding a sample of consultation responses. The use of publicly available data was resource efficient. Additionally, the use of all the available data ensured

For peer review only - http://bmjopen.bmj.com/site/about/guidelines.xhtml

that no perspectives were omitted, adding to internally validity. The omission of responses from individual members of the public was because most public responses lacked detail and were no more than a sentence long. Commercial influences on public health policy are unlikely to have changed over the past decade with no changes in lobbying rules or policy making procedures, making it highly likely that our findings from the 2007 consultation are applicable today.

There may be alternative methods by which the public influences policy making, such as by writing to their Member of Parliament. This is a study of only one case of public health policy making and our specific findings may not be generalisable to other aspects of dietary public health policy specifically or public health policy more generally. In this consultation, all members of a stakeholder category were treated as one, though there was some inter-category variation on position. A cross-question analysis could have been performed analysing responses by each question posed, although many of the responses were free text and did not address each question directly. In this study, we have only addressed what arguments and from whom are most influential in shaping public health policy, not specifically the various methods by which different stakeholders influence policy. There are also other ways by which interested parties could influence Ofcom, which we were unable to examine in this study. For example, Ofcom gave the option of providing confidential responses which were not available for us to incorporate into our dataset. Other informal lobbying may have occurred. Whether such channels of influence were used or whether similar arguments will have been used privately as were used publicly is unclear. Further work could explore other means of influence in due course.

Relationship to existing knowledge

Some literature exists on the methods by which public health advocates influence policy. In 2006, the New Zealand government held an 'Inquiry into Obesity' in order to determine what could be done to limit increasing obesity rates.[12] Jenkin *et al* found that in three out of four domains examined, the governmental position aligned with that of industry groups, with the exception being nutritional policy in schools. In the other three domains, national

For peer review only - http://bmjopen.bmj.com/site/about/guidelines.xhtml

obesity strategy, food industry policy, and advertising and marketing policy, the analysis determined that the governmental position allied with industry groups. Much like our study, public health groups were shown to have a limited impact on the eventual policies, with industry arguments proving more influential. An explanation suggested for this was the significance of the food industry to New Zealand's economy, highlighting how considerations outside of public health may importantly shape public health policy. It may be the case that similar factors shaped the eventual restrictions in our case study, despite the appearance of Ofcom wanting to develop 'proportionate restrictions', balancing commercial and public health interests. The question of what is proportionate appears to be determined by ideology and how much one feels government's role is to protect health even if it impacts on industry. If this is the case, we must question whether commercial companies can ever be truly motivated to improve health at the possible detriment to their short-term profits. A thematic analysis of alcohol industry documents in Australia[25] concluded that the industry attempted to create an impression of social responsibility whilst promoting interventions that did not affect their profits and campaigning against effective interventions that might affect profits. The *de facto* exemption of commercial stakeholders from bearing the negative external costs of their profitable endeavours (e.g. environmental, social or health impacts) has been widely questioned.[26]

Interpretation and implications of the study

Much of the research undertaken to date on stakeholder influences on public health policy has focused on industry behaviours and practices, whereas in this study we have treated both pro-industry and pro-public health groups equally in our analysis. Industry groups were apparently successfully able to argue that extensive restrictions would impact upon their commercial revenues, suggesting that their economic arguments importantly influenced the thinking of policy-makers. However, the future (external) costs of treating the potential health implications of HFSS food consumption did not appear to influence policy-making. This may be because any potential cost-savings are long-term and would apply to the health sector, for which Ofcom has no governmental responsibility, whereas the short-term costs would apply to the broadcast sector for which Ofcom is the regulatory body.

For peer review only - http://bmjopen.bmj.com/site/about/guidelines.xhtml

Public health advocacy is an activity in which many public health professionals are keen to become more effective to better ensure that evidence is translated into policy.[27,28] This study highlights that responding to public health policy consultations alone may not result in policy making favourable to public health and other avenues of influence may also need to be explored. Conversely, the change in the definition of children from 4-9 years to 4-15 years demonstrates that there is scope for public health advocates to shape policy should an issue be sufficiently clear and difficult to oppose. A more Machiavellian interpretation would be that to define children as aged 4-9 years at the outset may have been a cynical ploy aimed at ensuring that there was at least some ground to concede to public health stakeholders and distract from the more contentious issues. This is supported by the fact that the definition of children as aged 4-9 years was inherently questionable, given Ofcom's own definition of children as under 16 years, in line with the legal and medical definitions used in the UK. A few companies pointed to their media literacy campaigns as evidence that adolescents can understand advertising as an argument against redefining the scope of these restrictions to children aged 4-15 years. Evidence shows that advertisers simply use different ways to target adolescents,[29] rendering media literacy moot,[30] and suggesting that restrictions are still needed to protect adolescents.

The issue of TV advertising of less healthy foods remains highly politically sensitive and at the top of the public health strategy agenda for obesity.[18] Many UK public health organisations have recently campaigned to ban television advertising of less healthy foods before 9pm (the so-called 9pm watershed).[16,17,31–34] Our analysis of the 2006-7 consultation offers specific insights that could be influential in this ongoing national debate, in the same way as such analyses of historical documents have influenced tobacco control efforts in recent years.[10,35] The Ofcom regulation of television advertising of less healthy foods to children is one of few national public health policies of this sort to have been independently evaluated.[14,36] The independent evaluation found that the introduction of the regulations were not associated with a decrease in children's exposure to less healthy food advertising.[36] Our analysis sheds further light on why and how a regulatory policy that appears to have been ineffective in reducing children's exposure to less healthy food advertising came about. Publishing responses to public consultations in full is a key

For peer review only - http://bmjopen.bmj.com/site/about/guidelines.xhtml

component of transparent policy making. The UK Treasury's reluctance to make available responses to the Soft Drinks Industry Levy consultation is contrary to this principle.

Further questions and future research

How policy making is influenced through means other than public consultations should be further studied. Other means of applying political pressure such as political lobbying and having indirect relationships with positions of power are much more opaque and difficult to monitor. Parliamentary records of lobbying activity, copies of internal or leaked documents and registers of MPs interests may all be potential sources of data to explore these issues further. Interviews with former or current employees of policy forming bodies such as Ofcom, as well as other stakeholder groups could also be fruitful. Claims made during this consultation, such as industry claims of needing to issue profit warnings as a consequence of lost revenue from these restrictions, could be analysed. Thematic analysis of further documents such as the responses analysed in this study could provide valuable insight into whether a similar combination of commercial arguments and questioning scientific data is used across different public health policy consultations.

Conclusion

This analysis increases our understanding of how influential some stakeholders are in policy making and provides a framework from which further understanding of the influences on public health policy can be determined. From this case study, we can see that commercial influences on dietary public health policy-making appear to be somewhat greater than the influence of public health stakeholders in the initial framing of the consultation and this imbalance may have contributed to the ultimately compromised legislation. In this case, the potential for commercial impacts of legislation promoting public health appeared to outweigh the anticipated population health benefits in policy decision making.

Authors' contributions – The authors declare that they have no competing interests. Responses were coded by AR with a sub-sample independently duplicate coded by JA or MW. AR, JA and MW contributed to the manuscript in terms of both writing and editing.

For peer review only

Licensing Statement

The Submitting Author has the right to grant and does grant on behalf of all authors of the Work (as defined in the below author licence), an exclusive licence and/or a non-exclusive licence for contributions from authors who are: i) UK Crown employees; ii) where BMJ has agreed a CC-BY licence shall apply, and/or iii) in accordance with the terms applicable for US Federal Government officers or employees acting as part of their official duties; on a worldwide, perpetual, irrevocable, royalty-free basis to BMJ Publishing Group Ltd ("BMJ") its licensees and where the relevant Journal is co-owned by BMJ to the co-owners of the Journal, to publish the Work in BMJ Open and any other BMJ products and to exploit all rights, as set out in our licence.

The Submitting Author accepts and understands that any supply made under these terms is made by BMJ to the Submitting Author unless you are acting as an employee on behalf of your employer or a postgraduate student of an affiliated institution which is paying any applicable article publishing charge ("APC") for Open Access articles. Where the Submitting Author wishes to make the Work available on an Open Access basis (and intends to pay the relevant APC), the terms of reuse of such Open Access shall be governed by a Creative Commons licence – details of these licences and which Creative Commons licence will apply to this Work are set out in our licence referred to above.

For peer review only

References

1. Global Health Observatory. WHO | Obesity. WHO.

2. WHO | Reducing the impact of marketing of foods and non-alcoholic beverages on children. *WHO*. 2014. http://www.who.int/elena/titles/guidance_summaries/food_marketing_children/en/. Accessed August 8, 2017.

3. Kraak VI, Vandevijvere S, Sacks G, et al. Policy & practice Progress achieved in restricting the marketing of high-fat, sugary and salty food and beverage products to children. *Bull World Heal Organ*. 2016;94:540-548. doi:10.2471/BLT.15.158667.

4. Raine KD, Lobstein T, Landon J, et al. Restricting marketing to children: Consensus on policy interventions to address obesity. *J Public Health Policy*. 2013;34(2):239-253. doi:10.1057/jphp.2013.9.

5. Harris JL, Pomeranz JL, Lobstein T, Brownell KD. A Crisis in the Marketplace: How Food Marketing Contributes to Childhood Obesity and What Can Be Done. *Annu Rev Public Health*. 2009;30(1):211-225. doi:10.1146/annurev.publhealth.031308.100304.

6. Cairns G, Angus K, Hastings G, Caraher M. Systematic reviews of the evidence on the nature, extent and effects of food marketing to children. A retrospective summary. *Appetite*. 2013;62:209-215. doi:10.1016/j.appet.2012.04.017.

7. WHO | Protecting children from the harmful effects of food and drink marketing. *WHO*. 2014. http://www.who.int/features/2014/uk-food-drink-marketing/en/. Accessed June 28, 2017.

8. Sibbald B. Sugar industry sour on WHO report. *CMAJ*. 2003;168(12):1585. http://www.pubmedcentral.nih.gov/articlerender.fcgi?artid=156706&tool=pmcentrez&rendertype=abstract. Accessed December 23, 2015.

9. Savell E, Fooks G, Gilmore AB. How does the alcohol industry attempt to influence marketing regulations? A systematic review. *Addiction*. 2016;111(1):18-32. doi:10.1111/add.13048.

10. Savell E, Gilmore AB, Fooks G, Weishaar H, Gilmore A. How Does the Tobacco Industry Attempt to Influence Marketing Regulations? A Systematic Review. Derrick

For peer review only

For peer review only - http://bmjopen.bmj.com/site/about/guidelines.xhtml

GE, ed. *PLoS One*. 2014;9(2):e87389. doi:10.1371/journal.pone.0087389.

11. Grüning T, Gilmore AB, McKee M. Tobacco industry influence on science and scientists in Germany. *Am J Public Health*. 2006;96(1):20-32. doi:10.2105/AJPH.2004.061507.

12. Jenkin G, Signal L, Thomson G. Nutrition policy in whose interests? A New Zealand case study. *Public Health Nutr*. 2012;15(8):1483-1488. doi:10.1017/S1368980011003028.

13. Adams J, Tyrrell R, Adamson AJ, White M. Effect of restrictions on television food advertising to children on exposure to advertisements for “less healthy” foods: repeat cross-sectional study. *PLoS One*. 2012;7(2):e31578. doi:10.1371/journal.pone.0031578.

14. Adams J, Hennessy-Priest K, Ingimarsdóttir S, Sheeshka J, Ostbye T, White M. Food advertising during children’s television in Canada and the UK. *Arch Dis Child*. 2009;94(9):658-662. doi:10.1136/adc.2008.151019.

15. Children’s Food Campaign. *Through the Looking Glass: A Review of Topsy-Turvy Junk Food Marketing Regulations*.; 2013. https://www.sustainweb.org/publications/through_the_looking_glass/. Accessed October 10, 2017.

16. Sugar Reduction The evidence for action. 2015. www.gov.uk/phe. Accessed October 10, 2017.

17. The Labour Party. Labour Manifesto. http://www.labour.org.uk/index.php/manifesto2017. Published 2017. Accessed June 28, 2017.

18. *Childhood Obesity: A Plan for Action Chapter 2 2 DH ID Box Title: Childhood Obesity: A Plan for Action, Chapter 2*.; 2018. www.nationalarchives.gov.uk/doc/open-government-licence/. Accessed November 27, 2018.

19. O’Brien BC, Harris IB, Beckman TJ, Reed DA, Cook DA. Standards for reporting qualitative research: a synthesis of recommendations. *Acad Med*. 2014;89(9):1245-1251. doi:10.1097/ACM.0000000000000388.

For peer review only

For peer review only - http://bmjopen.bmj.com/site/about/guidelines.xhtml

20. Ofcom. *Television Advertising of Food and Drink Products to Children: Final Statement*.

21. Hsieh H-F, Shannon SE. Three approaches to qualitative content analysis. *Qual Health Res*. 2005;15(9):1277-1288. doi:10.1177/1049732305276687.

22. Gale NK, Heath G, Cameron E, Rashid S, Redwood S. Using the framework method for the analysis of qualitative data in multi-disciplinary health research. *BMC Med Res Methodol*. 2013;13:117. doi:10.1186/1471-2288-13-117.

23. Ofcom. Television Advertising of Food and Drink Products to Children - Ofcom. https://www.ofcom.org.uk/consultations-and-statements/category-2/foodads_new. Accessed August 8, 2017.

24. Department of Health. *Choosing Health: Making Healthy Choices Easier*.; 2004. https://webarchive.nationalarchives.gov.uk/20120509221645/http://www.dh.gov.uk/prod_consum_dh/groups/dh_digitalassets/@dh/@en/@ps/documents/digitalasset/dh_133489.pdf. Accessed March 12, 2019.

25. Miller PG, de Groot F, McKenzie S, Droste N. Vested interests in addiction research and policy. Alcohol industry use of social aspect public relations organizations against preventative health measures. *Addiction*. 2011;106(9):1560-1567. doi:10.1111/j.1360-0443.2011.03499.x.

26. Stuckler D, Nestle M. Big food, food systems, and global health. *PLoS Med*. 2012;9(6):e1001242. doi:10.1371/journal.pmed.1001242.

27. Dorfman L, Krasnow ID. Public Health and Media Advocacy. *Annu Rev Public Health*. 2014;35(1):293-306. doi:10.1146/annurev-publhealth-032013-182503.

28. Herrick C. The post-2015 landscape: vested interests, corporate social responsibility and public health advocacy. *Sociol Health Illn*. 2016;38(7):1026-1042. doi:10.1111/1467-9566.12424.

29. Livingstone S, Helsper EJ. Does Advertising Literacy Mediate the Effects of Advertising on Children? A Critical Examination of Two Linked Research Literatures in Relation to Obesity and Food Choice. *J Commun*. 2006;56(3):560-584. doi:10.1111/j.1460-2466.2006.00301.x.

For peer review only

For peer review only - http://bmjopen.bmj.com/site/about/guidelines.xhtml

30. Montgomery KC, Chester J. Interactive Food and Beverage Marketing: Targeting Adolescents in the Digital Age. *J Adolesc Heal*. 2009;45(3):S18-S29. doi:10.1016/j.jadohealth.2009.04.006.

31. Sustain / Children's Food Campaign. Junk Food Marketing. https://www.sustainweb.org/childrensfoodcampaign/junk_food_marketing/. Accessed November 1, 2017.

32. Cancer Research UK. Being overweight or obese could cause around 700,000 new UK cancers by 2035 | Cancer Research UK. http://www.cancerresearchuk.org/about-us/cancer-news/press-release/2016-01-07-being-overweight-or-obese-could-cause-around-700000-new-uk-cancers-by-2035. Accessed November 1, 2017.

33. Obesity Health Alliance. Tackling Childhood Obesity - 2017 Election Manifesto. http://obesityhealthalliance.org.uk/policy/. Accessed November 1, 2017.

34. Faculty of Public Health. Start Well, Live Better. http://www.fph.org.uk/start_well%2C_live_better_-_a_manifesto. Accessed November 1, 2017.

35. Fooks GJ, Gilmore AB. Corporate philanthropy, political influence, and health policy. *PLoS One*. 2013;8(11):e80864. doi:10.1371/journal.pone.0080864.

36. Adams J, Tyrrell R, Adamson AJ, White M. Effect of restrictions on television food advertising to children on exposure to advertisements for "less healthy" foods: repeat cross-sectional study. *PLoS One*. 2012;7(2):e31578. doi:10.1371/journal.pone.0031578.

For peer review only

Figure titles and legends

Figure 1: A timeline of the Ofcom process on developing new recommendations for limiting television food advertising to children.

Figure 1 legend:

* ’Interested parties’ are stakeholder groups who may have been affected by the proposed changes, including advertising agencies, advocacy groups, broadcasters, charities, healthcare associations, politicians, the food industry and the general public.

Table 1: Packages of regulations proposed by Ofcom in the initial consultation (March 2006)

Table 1 legend:

* HFSS food = High, Fat, Sugar and Salt foods

+ ‘of particular appeal to children’ = when the proportion of people watching who are children is more than 120% of the proportion of children in the UK population[23]

Table 2: The categories into which stakeholder groups were classified. A list of each group classified by category can be found in the Appendix.

Table 3: The changes in Ofcom’s position during the course of the consultation

Appendix

Table A: The questions Ofcom asked as part of the consultation

Table B1: The classification of the responses by organisational category

Table B2: The classification of the responses by organisational category (continued)

For peer review only - http://bmjopen.bmj.com/site/about/guidelines.xhtml

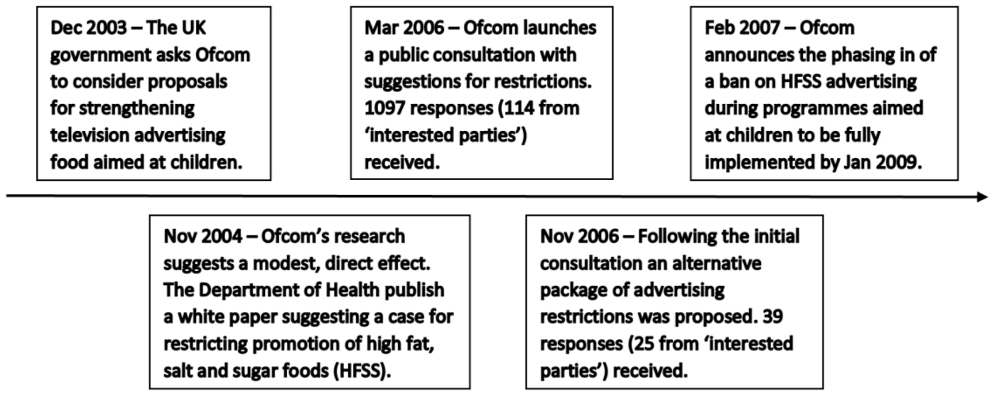

Figure 1: A timeline of the Ofcom process on developing new recommendations for limiting television food advertising to children.

Legend: * 'Interested parties' are stakeholder groups who may have been affected by the proposed changes, including advertising agencies, advocacy groups, broadcasters, charities, healthcare associations, politicians, the food industry and the general public.

Question 1	Do you agree that the regulatory objectives set out in paragraph 5.2 above are appropriate?
Question 2	Do you consider that it is desirable to distinguish between foods that are high in fat, salt or sugar and those that are healthier in order to achieve the regulatory objectives, or could an undifferentiated approach provide a reasonable alternative?
Question 3	If so, do you consider the FSA's nutrient profiling scheme to be a practical and reasonable basis for doing so? If not, what alternative would you propose? (Note: The nutrient profiling scheme was developed by the FSA and handed to Ofcom following extensive consultation (see FSA web site). This being the case, and given the scheme itself and the science upon which it is based fall outside Ofcom's area of responsibility and expertise, it is not appropriate in this consultation to seek responses on those matters)
Question 4	Do you agree that voluntary self-regulation would not be likely to meet Ofcom's regulatory objectives or the public policy objectives?
Question 5	Do you agree that the exclusion of all HFSS advertising before 9.00pm would be disproportionate?
Question 6	Do you agree that all food and drink advertising and sponsorship should be excluded from programmes aimed at pre-school children?
Question 7	Do you agree that revised content standards should apply to the advertising or sponsorship of all food and drink advertisements?
Question 8	Do you consider that the proposed age bands used in those rules aimed at preventing targeting of specific groups of children are appropriate?
Question 9	Do you consider the proposed content standards including their proposed wording to be appropriate, and if not, what changes would you propose, and why?
Question 10	Do you consider a transitional period would be appropriate for children's channels in the context of the scheduling restrictions, and if so, what measure of the 'amount' of advertising should be used?
Question 11	Do you consider there is a case for exempting low child audience satellite and cable channels from the provisions of Package 3?
Question 12	Do you agree that there should not be a phase-in period for children's channels under Package 3?
Question 13	Which of the three policy packages would you prefer to be incorporated into the advertising code and for what reasons?
Question 14	Alternatively, do you consider that a combination of different elements of the three packages would be suitable? If so, which elements would you favour within an alternative package? (You should note that the analysis in the Impact Assessment has focused on estimating the costs of restricting scheduling, volume, and content separately and would therefore allow consideration of other combinations of the same elements).
Question 15	Where you favour either Package 1 or 2, do you agree that it would be appropriate to allow children's channels a transitional period to phase in restrictions on HFSS / food advertising, on the lines proposed?
Question 16	Do you consider that the packages should include restrictions on brand advertising and sponsorship? If so, what criteria would be most appropriate to define a relevant brand? If not, do you see any issue with the prospect of food manufacturers substituting brand advertising and sponsorship for product promotion?
Question 17	Ofcom invites comments on the implementation approach set out in paragraph 5.45 and 5.46.

Advertising stakeholders	Broadcast stakeholders	Lawyers	Politicians	Retailers
Institute of Practitioners in Advertising	Producer's Alliance for Cinema and Television 1	Baker and McKenzie LLP	Mary Creagh MP	Sainsbury
Advertising Association	Five		Welsh Assembly	The Co-operative
Incorporated Society of British Advertisers 1	Channel 4		All Party Parlimentary group on Heart Disease	British Retail Consortium 1
Mediavest Manchester	Flextech television		David Amess MP	British Retail Consortium 2
Zenith Optimedia	ITV			
Mindshare	GMTV			
Incorporated Society of British Advertisers 2	Jetix, Nickelodeon and Turner			
	Producer's Alliance for Cinema and Television 2			
	Broadcast Advertising Clearance Centre			
	British Academy of Film and Television Arts			
	Broadcast Committee of Advertising Practice			

For peer review only - http://bmjopen.bmj.com/site/about/guidelines.xhtml

Food companies	Food industry representative groups	Civil society groups	Public health stakeholders
Kellogg 1	Food Advertising Unit 1	Kids Inc	National Heart Forum 1
Kraft 1	Food and Drink Federation 2	Consumer Council of Northern Ireland	Northern Ireland Chest Heart and Stroke 2
Pepsico	Snack Nut and Crisp Manufacturers Association 1	Children's Food Bill Coalition	UK Public Health Association
GlaxoSmithKline 1	Food Advertising Unit 2	National Consumer Council 1	Royal Society of Health
United Biscuits	Food and Drink Federation 1	Which 1	Consensus Action on Salt and Health
Nestle	Dairy Council	Officer of the Children's Commissioner	Nutrition Society
Cadbury	Snack Nut and Crisp Manufacturers Association 2	School Food Trust 1	Diabetes UK
Ferrero 1	Provision Trade Federation	Trading Standards Institute	Association for the Study of Obesity
Masterfoods 2	British Cheese Board	Which 2	Heart of Mersey 2
Coca-cola 1	Biscuit Cake Chocolate Confectionary Association 1	Welsh Consumer Council	National Oral Health Promotion Group
McDonalds 2	Biscuit Cake Chocolate Confectionary Association 2	Food Aware	Scientific Advisory Committee on Nutrition
Vimto	Dairy UK	Safefood Ireland	British Psychological Society
Wrigley		The Caroline Walker Trust	British Dietetic Association
Wiltshire farm foods		Advisory Committee for England	National Heart Forum 2
Unilever		Voice of the Listener and Viewer 2	British Heart Foundation
GlaxoSmithKline 2		Advertising Advisory Committess	British Medical Association 1
Coca-cola 2		British Nutrition Foundation	Cheshire and Merseyside Public Health Network
Masterfoods 2		Food Ethics Council	Health Protection Agency Northern Ireland
Kraft 2		Voice of the Listener and the Viewer 1	Irish Heart Foundation 1
McDonalds 1		National Consumer Council 2	National Heart Alliance Ireland 1
RHM Group		National Family and Parenting Institute	National Heart Alliance Ireland 2
Kellogg 2		National Union of Teaachers	International Association for the Study of Obesity 1
Ferrero 2		The Nutrition Society	British Medical Association 2
		Children's Food Campaign	Heart of Mersey 1
		Consumer Council	Northern Ireland Chest Heart and Stroke 1
		Barnardos	Irish Heart Foundation 2
		National Children's Bureau	NHS Borders
		Public Voice	Medical Research Council 1
		School Food Trust	British Heart Foundation 2
		Scotland's Commissioner for Young People	Cancer Research UK
		Food Standards Agency	Northern Ireland Chest Heart and Stroke 3
		National Youth Agency	International Association for the Study of Obesity 2
		Advisory Committee for Northern Ireland	Royal College of Physicians
		Food Commission 1	Weight Concern
		Women's Institute 1	British Dental Association
		The Food Commission	Medical Research Council 2
		The Obesity Awareness and Solutions Trust	Joint statement by the British Heart Foundation, Cancer Research UK and Diabetes UK
		National Federation of Women's Institutes 1	Royal College of Nursing
		National Federation of Women's Institutes 2	

eer review only - http://bmjopen.bmj.com/site/about/guidelines.

Reporting checklist for qualitative study.

Based on the SRQR guidelines.

Instructions to authors

Complete this checklist by entering the page numbers from your manuscript where readers will find each of the items listed below.

Your article may not currently address all the items on the checklist. Please modify your text to include the missing information. If you are certain that an item does not apply, please write "n/a" and provide a short explanation.

Upload your completed checklist as an extra file when you submit to a journal.

In your methods section, say that you used the SRQR reporting guidelines, and cite them as:

O'Brien BC, Harris IB, Beckman TJ, Reed DA, Cook DA. Standards for reporting qualitative research: a synthesis of recommendations. Acad Med. 2014;89(9):1245-1251.

		Reporting Item	Page Number
	#1	Concise description of the nature and topic of the study identifying the study as qualitative or indicating the approach (e.g. ethnography, grounded theory) or data collection methods (e.g. interview, focus group) is recommended	5
	#2	Summary of the key elements of the study using the abstract format of the intended publication; typically includes background, purpose, methods, results and conclusions	2
Problem formulation	#3	Description and signifcance of the problem / phenomenon studied: review of relevant theory and empirical work; problem statement	4
Purpose or research question	#4	Purpose of the study and specific objectives or questions	5
Qualitative approach and research paradigm	#5	Qualitative approach (e.g. ethnography, grounded theory, case study, phenomenolgy, narrative research) and	5

For peer review only

		guiding theory if appropriate; identifying the research paradigm (e.g. postpositivist, constructivist / interpretivist) is also recommended; rationale. The rationale should briefly discuss the justification for choosing that theory, approach, method or technique rather than other options available; the assumptions and limitations implicit in those choices and how those choices influence study conclusions and transferability. As appropriate the rationale for several items might be discussed together.	
Researcher characteristics and reflexivity	#6	Researchers' characteristics that may influence the research, including personal attributes, qualifications / experience, relationship with participants, assumptions and / or presuppositions; potential or actual interaction between researchers' characteristics and the research questions, approach, methods, results and / or transferability	1
Context	#7	Setting / site and salient contextual factors; rationale	4
Sampling strategy	#8	How and why research participants, documents, or events were selected; criteria for deciding when no further sampling was necessary (e.g. sampling saturation); rationale	5
Ethical issues pertaining to human subjects	#9	Documentation of approval by an appropriate ethics review board and participant consent, or explanation for lack thereof; other confidentiality and data security issues	8
Data collection methods	#10	Types of data collected; details of data collection procedures including (as appropriate) start and stop dates of data collection and analysis, iterative process, triangulation of sources / methods, and modification of procedures in response to evolving study findings; rationale	5
Data collection instruments and technologies	#11	Description of instruments (e.g. interview guides, questionnaires) and devices (e.g. audio recorders) used for data collection; if / how the instruments(s) changed over the course of the study	6
Units of study	#12	Number and relevant characteristics of participants, documents, or events included in the study; level of	8

For peer review only

		participation (could be reported in results)	
Data processing	#13	Methods for processing data prior to and during analysis, including transcription, data entry, data management and security, verification of data integrity, data coding, and anonymisation / deidentification of excerpts	6
Data analysis	#14	Process by which inferences, themes, etc. were identified and developed, including the researchers involved in data analysis; usually references a specific paradigm or approach; rationale	6
Techniques to enhance trustworthiness	#15	Techniques to enhance trustworthiness and credibility of data analysis (e.g. member checking, audit trail, triangulation); rationale	7
Syntheses and interpretation	#16	Main findings (e.g. interpretations, inferences, and themes); might include development of a theory or model, or integration with prior research or theory	8
Links to empirical data	#17	Evidence (e.g. quotes, field notes, text excerpts, photographs) to substantiate analytic findings	11
Intergration with prior work, implications, transferability and contribution(s) to the field	#18	Short summary of main findings; explanation of how findings and conclusions connect to, support, elaborate on, or challenge conclusions of earlier scholarship; discussion of scope of application / generalizability; identification of unique contributions(s) to scholarship in a discipline or field	17
Limitations	#19	Trustworthiness and limitations of findings	18
Conflicts of interest	#20	Potential sources of influence of perceived influence on study conduct and conclusions; how these were managed	1
Funding	#21	Sources of funding and other support; role of funders in data collection, interpretation and reporting	1

The SRQR checklist is distributed with permission of Wolters Kluwer © 2014 by the Association of American Medical Colleges. This checklist was completed on 26. November 2018 using http://www.goodreports.org/, a tool made by the EQUATOR Network in collaboration with Penelope.ai

For peer review only
